# Strategies to Facilitate Service Utilization Among Youth at Risk for HIV: A Randomized Controlled Trial (ATN 149)

**DOI:** 10.1007/s10461-024-04545-2

**Published:** 2024-11-28

**Authors:** Dallas Swendeman, Mary Jane Rotheram-Borus, Elizabeth Mayfield Arnold, Maria Isabel Fernández, W. Scott Comulada, Kelsey Ishimoto, William Gertsch, Debra A. Murphy, Manuel Ocasio, Sung-Jae Lee, Katherine A. Lewis, The Adolescent HIV Medicine Trials Network (ATN) CARES Team, Sue Ellen Abdalian, Robert Bolan, Yvonne Bryson, Antwon Chaplin, Ruth Cortado, Catherine Donahue, Naihua Duan, Risa Flynn, Jasmine Fournier, Sergio Jimenez, Tara Kerin, Jeffrey Klausner, Jody Kussin, Marguerita Lightfoot, Norweeta Milburn, Jasmine Mosafer, Aaron Moses, Karin Nielsen, Wilson Ramos, Cathy J. Reback, Panteha Hayati Rezvan, Wenze Tang, Yara Tapia, Demi Thomas, Stacey Urauchi, Robert E. Weiss

**Affiliations:** 1 Semel Institute for Neuroscience and Human Behavior, University of California, 760 Westwood Plaza, Suite 37-360A, Los Angeles, CA 90024 USA; 2 Department of Psychiatry, University of Kentucky, Lexington, USA; 3 College of Osteopathic Medicine, Nova Southeastern University, Fort Lauderdale, FL USA; 4 Department of Pediatrics, Tulane University School of Medicine, New Orleans, LA USA

**Keywords:** Youth, Support services, HIV prevention, LGBTQ+, Text-messaging, Peer support, Strengths-based coaching

## Abstract

Sexual and gender minority youth (SGMY) are vulnerable to acquiring HIV and need HIV prevention and health services, but may have competing needs. A prior analysis found that PrEP use reports increased in a combination intervention study arm with coaching, peer support, and automated text-messages. This paper examines ancillary support and healthcare services utilization as secondary intervention objectives. SGMY (N = 895, 40% Black, 29% Latino) in Los Angeles and New Orleans were recruited from May, 2017 to August, 2019 and randomized to four intervention conditions: (a) automated text-messaging and monitoring intervention (AMMI), (b) AMMI plus peer support online (AMMI+PS), (c) AMMI plus strengths-based coaching by near-peer paraprofessionals (AMMI+C), or (d) all three (AMMI+PS+C). Intent-to-treat multivariate regression analyses evaluated the interventions’ efficacy on past 4-month reports of ancillary support services use, having a regular healthcare provider, receiving care from doctor’s office or clinic and mental health specialists, and participation in mental health support groups and HIV prevention programs. Ancillary services utilization reports declined from 40% of youth reporting an average of 4.4 services at baseline to 22.6% reporting 2.5 services by 24 months. Food, housing, transportation, and other basic services were utilized most frequently. Youth in the two coaching interventions maintained higher reports of services use over time compared to AMMI-only (both OR 1.23, 95%CI 1.12–1.35) and to AMMI+PS (both OR 1.20 95%CI 1.08–1.33). Our coaching intervention may support SGMY to stay engaged in support services. Results may be limited by self-report biases. It is unclear if these services are related to better long-term outcomes.

**Supplementary Information:**

The online version contains supplementary material available at 10.1007/s10461-024-04545-2.

## Introduction

Sexual and gender minority persons represent a disproportionate share of the over 38,000 new HIV cases in the United States as of 2022, with younger, Black, and Latino persons over-represented among new infections. [1]. Ending the HIV epidemic through prevention and treatment continuums that include biomedical, behavioral, and ancillary support services is a top public health priority [1–4]. Competing needs and priorities of sexual and gender minority youth (SGMY) may make engaging in HIV prevention and support services difficult.

SGMY may experience a cascade of structural and behavioral vulnerabilities to HIV and other health disparities linked to intersections of minoritized identities and discrimination. For example, when SGMY disclose their sexual or gender identities to their families, they may experience familial rejection and ejection from their homes [5, 6]. Experiencing stigma, discrimination and unstable housing may increase risks for substance use and mental health problems, unemployment, sex work, criminal justice contact and incarceration, and HIV [7–10]. The resulting syndemic challenges increase vulnerability to HIV [11–14] while also presenting barriers to accessing and utilizing healthcare, prevention, and support services [15–18]. Transgender and gender diverse youth may also seek and prioritize gender-affirming care while experiencing stigma-related barriers to accessing other care and HIV prevention services [15, 19]. Yet, several studies with youth and SGMY identify low utilization of healthcare and support services [13, 14, 18].

Some youth vulnerable to HIV infection may have difficulty traversing systems-of-care due to a range of challenges. They may not know how to start seeking services, lack trust in organizations or individuals who might provide assistance, or face practical (e.g., insurance/costs, transportation, inconvenient service hours or location) and socio-emotional (e.g., confidentiality concerns, stigma) barriers to accessing care [10, 20]. Strategies for supporting SGMY to be aware, motivated, and able to navigate a range of diffuse service systems must be identified to reduce SGMY’s barriers to access and to maintain consistent engagement in HIV prevention, healthcare and ancillary support services.

The current study was a randomized controlled trial that aimed to increase HIV prevention continuum engagement among SGMY, with previously reported primary outcomes of prevention option choices; PrEP use, condom use, Post-Exposure Prophylaxis (PEP) or partner strategies (i.e., reducing sexual partner numbers) [21]. Secondary outcomes included substance use, mental health, and housing security. Given the multiple challenges facing SGMY, the study and interventions were designed to concurrently address healthcare and support services utilization through referrals by interviewers and automated texting as an enhanced standard of care, peer support with service referrals and discussions in an online mobile-web discussion board, and a strengths-based coaching intervention with services referral and navigation, goal setting, follow-up and problem solving delivered by phone or in-person with texting follow-up. Intervention details and rationales are provided in prior protocol papers and briefly below [22–26]. The primary outcome paper reports significantly higher increases in reports of PrEP use, but not other outcomes, in the study arm combining all three intervention strategies compared to enhanced standard of care control-comparison arm with interviewer delivered referrals, HIV and STI testing, and automated text-messaging [21]. This paper presents secondary analyses of healthcare and support services utilization over time by intervention arm among SGMY.

## Methods

### Intervention Design: Disruptive Innovations in HIV Prevention and Support

We used a “Disruptive Innovations” [27] approach in our intervention design and delivery, based on common factors, practices, principles, theories and strategies found in content analyses of efficacious HIV prevention interventions for youth [28–31]. The Disruptive Innovations approach in healthcare and prevention focuses attention on core, evidence-based functions delivered by simpler and “good enough” modalities that can reach the most people in need at lower costs for design, adoption, adaptation, and delivery relative to specialized services designed for the highest need or most complex clients or patients [27, 29]. Common examples in healthcare are $2 eyeglasses, “minute clinics” in pharmacies, community health workers, mobile health, and telemedicine [27, 32].

At study conception (application) in January 2016, there were no evidenced-based interventions for PrEP use among youth, but there was a large evidence-base for efficacy of behavioral interventions for other HIV prevention and related outcomes for youth, which had been synthesized and analyzed for common components to inform future intervention design [28–31]. Our main innovations were in using digital technology delivery modalities of automated texting, online discussion boards for peer support, and telehealth option for coaching in addition to in-person, building off a nascent evidence base from work with similar populations and the promise of digital technologies’ reach and engagement given the overwhelming majority 13 to 17 year olds having cell phones at the time (88% in 2015) with daily use [33]. We also explicitly selected intervention modalities that did not rely on smartphones or apps, instead relying on simpler, easier to adapt and sustain, text-messaging and phone calls. In addition, the advent of biomedical HIV prevention (PrEP) precipitated a lack of ongoing funding and implementation support for efficacious but complex, multi-session behavioral interventions in the Center for Disease Control and Prevention’s (CDC) transition from the Diffusion of Effective Behavioral Interventions (DEBI) project [34] to the High Impact HIV Prevention (HIP) program focused on capacity building for PrEP delivery [35].

Our second primary innovation was in response to our and others’ experiences working with the CDC’s Replicating Effective Programs (REP) and DEBI project trainings with implementer feedback on difficulties delivering EBIs with fidelity to scripted, multi-session, structured activity manuals [36–38]. Therefore, we designed a non-manualized and unscripted coaching intervention, based on the strengths-based case management model [39] and common evidence-based practice elements [38] delivered by near peer paraprofessionals in person, by phone, and text-message. We provide brief information on interventions below and details are provided in several prior protocol and outcomes papers [21–26].

#### Automated Text-Messaging and Monitoring Intervention (AMMI)

AMMI was offered to all participants as part of enhanced standard of care to provide an ethically required intervention for youth. The intervention was adapted primarily from our experience collaborating on Project Tech Support, which demonstrated efficacy of automated texting to reduce HIV risk behaviors and improve medication adherence among gay and bisexual men using methamphetamine [40], anticipated to be an overlapping population in our sample. Libraries of more than 400 theory-based messages were initially adapted and then updated at study mid-point by peer and professional staff and youth advisory boards. Up to five automated, unidirectional, informational, motivational, behavioral, reminder and service linkage messages were sent daily in five content streams on physical health and health care, mental health and wellness, sexual health, substance use, and medication reminders. In addition, a weekly self-monitoring survey was sent via text or email with 7 questions related to primary and secondary outcomes (e.g., number of days having sex without condoms, experience depression or anxiety, housing or food insecurity), with $1 incentive provided for each completed survey.

#### Peer Support Groups Online

This intervention was adapted primarily from our colleague’s experiences with Project Hope, which demonstrated promising feasibility, acceptability and efficacy for peer-led HIV prevention on social media groups among African American and Latino men who have sex with men [41, 42], also anticipated to be an overlapping study population. Peer support also had a promising evidence-base in HIV more broadly [43]. Our online group modality was also informed by our prior experiences with in-person small group HIV prevention interventions for youth, in which we encountered significant scheduling, transportation, and confidentiality barriers to participation [44]. Two intervention arms were offered peer support, with participants modestly incentivized at $10 per week for posting three messages for up to 16 weeks as mutual peer supporters on a private social media platform (Muut, similar to Discord or Reddit). The near peer coach study staff moderated, seeded discussions, shared realistic experiences, solutions, and services information, and corrected misinformation with evidence-based information. Participants shared their opinions, experiences, and recommendations for services in their communities.

#### Strengths-Based Coaching

This intervention was adapted from the strengths-based case management model previously used with youth in high-risk situations [39]. We incorporated 14 common evidence-based practice elements for youth interventions applied to multiple domains described briefly below (also see Supplemental Figs. 1 & 2) [28, 38]. Coaches engaged youth in an initial strengths assessment on domains of daily living (housing, food and economic security), social relationships, healthcare, physical health, mental health, and risks of substance use and sexual health. Strengths and challenges were identified, and up to three youth-driven goals set, including for HIV prevention and indicated services and linkages with warm handoffs rather than providing passive referral information. Follow-up sessions reviewed goal progress, problem solved what did or did not work and reset goals. Although primarily delivered via phone calls, the delivery was flexible to allow in-person meetings and brief follow-ups by text-message. Coaching was available as needed, initially weekly and then monthly, over the 24-month follow-up period to meet needs of developmental transitions, crises and changing life circumstances. Coaches with prior experience as frontline HIV prevention workers (e.g., PrEP navigators, peer educators) were hired and trained in the strengths assessment and practice elements with weekly group supervision.

This paper focuses on the relative efficacy of these increasingly costly and burdensome intervention strategies to support service utilization. SGMY were randomized to four conditions: 1) enhanced standard of care with AMMI; (2) AMMI plus peer support (AMMI+PS); (3) AMMI plus coaching (AMMI+C); and (4) AMMI plus peer support plus coaching (AMMI+PS+C). We hypothesized that the interventions would have synergistic rather than additive effects given their complementary functions of informational and motivational prompts, peer social support and norming, and goal-focused coaching [22].

All procedures were approved by the University of California, Los Angeles Institutional Review Board (IRB#16-001674-AM-00005) and ATN Study Monitoring Committee.

### Recruitment & Eligibility

Youth were recruited from 13 community-based and healthcare agencies in neighborhoods with high HIV seroprevalences in Los Angeles and New Orleans from May 2017 to August 2019. The primary study population was SGMY ages 12 to 24, eligible to participate if they reported being gay, bisexual and other cisgender men who have sex with men, transgender women and men, and gender diverse or non-binary youth. These youth were eligible without requirements for current or lifetime HIV risk behaviors at baseline to include a broad age range of adolescents who had not initiated or had intermittent sexual behaviors, and anticipating developmental transitions over the 24-month follow-up or potential under-reporting at screening. Eligibility did not require cell phone ownership and recruiters were trained to provide support for acquiring Universal Lifeline Program phones with wireless access if participants did not have their own cell phones, although need was rare at screening.

**Fig. 1:**
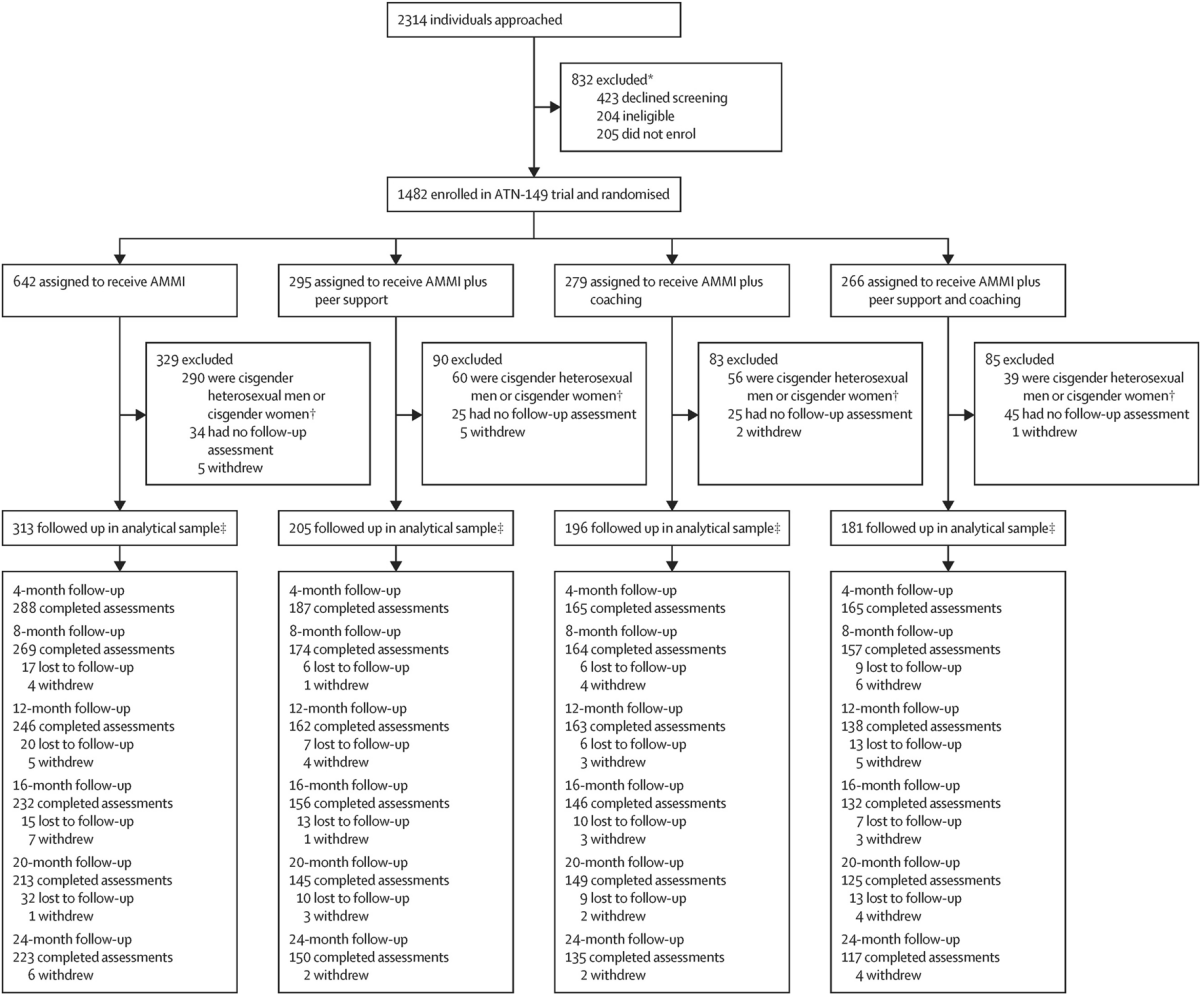
CONSORT enrollment and retention diagram for adolescent trials network (ATN) 149

The primary outcome paper [21] reports details of screening and enrollment shown in Fig. 1 (Consort Enrollment and Retention Diagram). Briefly, 2314 youth were approached to be screened, 173 declined screening, 250 were identified as HIV seropositive and triaged to other studies, and 205 eligible youth declined enrollment and baseline assessment. Initially, cis-gender female and heterosexual male youth were eligible based on an algorithm of behavioral and structural risk factors (e.g., substance use, sexual behaviors, incarceration or probation, or homeless histories) but a funder-initiated study protocol change executed in January 2019 excluded these youth from future eligibility or follow-up past 12 months for those already enrolled (n = 445), except for heterosexually identified cis-gender male youth reporting sex with men or testing positive for rectal STI at baseline or follow-ups (n = 43). Thus, 1037 SGMY were eligible, enrolled and randomized after baseline to either AMMI, AMMI+PS, AMMI+C, or AMMI+PS+C for the primary study aims. The analytic sample (n = 895) is composed of randomized SGMY who completed at least one follow-up assessment and, therefore, provide information on intervention efficacy over time. Participants were reassessed at six follow-up points at four-month intervals over 24 months through November 2021 with 90%–70% retention.

### Assessments

Interviewers verbally administered screening and study assessments, entering responses in the study mobile-web assessment, intervention, and case management platform, CommCare by Dimagi Inc. [23]. Interviewer-Recruiters were similar in age, race/ethnicity and SGM status to the sample and typically held a bachelor’s degree. Each was deemed as competent in the screening and interviewing procedures by supervisors, certified in phlebotomy and HIV testing, and received weekly in-service trainings and supervision. Study visits also included rapid HIV testing, biomarkers for substance use, and rapid PCR testing for sexually transmitted infections (STIs) with same day treatment and partner therapy for bacterial STIs [26].

After each assessment, interviewers provided general and specifically indicated service referral information via resource guides but did not call to facilitate any referral. All participants also received AMMI as minimal ethically required intervention for youth. Thus, the study procedures reflected an enhanced standard of care of repeat assessments, passive referrals, automated texting, and HIV/STI testing and treatment. Participants were provided $50 for completing each assessment. Retention was supported by phone, text, email, social media, and family or friend contacts as well as in-person presence by interviewers at recruitment sites.

### Outcomes

At each assessment, participants reported support services utilization over the past 4 months as use of each of the following (yes/no): housing; food; clothing; toiletries and hygiene products; transportation (including taxi, token, mileage reimbursement, etc.); employment services; case management; mental health counseling or treatment, substance use counseling or drug treatment, healthcare insurance counseling; healthcare service navigation (Peer navigation, HIV navigator, etc.); hormone therapy/hormone therapy counseling; post-incarceration or parole services; child care, or other services (which youth specified and interviewers recorded as open ended text). Other responses were recoded to listed options when appropriate. Given the diversity of youth and their needs, we created a binary yes–no indicator for any support service utilization over the previous four months.

We also examined four outcomes from questions specifically focused on healthcare utilization and another on HIV prevention program participation. Healthcare was assessed with two questions, “Do you currently have a health care provider, a doctor or a clinic that you can go to if you need care?” (yes/no) and “In the past 4 months, how many times did you receive care from a doctor's office, clinic or wellness center?” The latter count response was dichotomized 0 vs. 1+for analyses to reflect primary care and sexual health visit standards of care ranging from annually to quarterly, respectively, and to mitigate impact of outliers with high numbers of healthcare visits for chronic conditions or acute episodes. Mental health care was assessed with how many times “receive outpatient care from a mental health specialist like a counselor, social worker or psychiatrist?” and “receive or participate in any self-help mental health services such as a mental health support group for anxiety or depression?” The sixth outcome was how many times “…participate in any HIV prevention program/workshop/event provided by any community-based organization, peer-support group, online group or any other organizations or individuals?” These three “how many times” count question response values were winsorized (capped) at 20 based on examination of response distribution tails and to reflect approximately weekly counseling, support group, or HIV prevention program session participation over the recent 4-month or 17 week recall periods and to mitigate outlier effects (e.g., a few responses indicated daily or near daily participation, likely reflecting digital health intervention participation).

### Covariates

We included descriptive information (Table 1) and covariates in adjusted analyses with variables on key demographic characteristics, that had baseline study arm imbalances, that may be associated with services use, and were associated with loss to follow up. Table 1 shows responses as originally assessed with the following clarifications. Race was assessed separately from Latino/Hispanic ethnicity and then recoded for race/ethnicity in Table 1. Income was assessed with the question, “How much money, from all sources combined, did you receive last month? Include money received formally and informally from a job, legally, illegally, under the table, from disability, public assistance, or any other sources.” Due to skewness in responses, continuous responses were recoded to binary indicator for above or below 2021 federal poverty level. Hazardous alcohol drinking was assessed with AUDIT-C score 4 or higher for males and 3+for females (assigned at birth). Substance use was assessed with the question, “Have you used in the past 4 months. (yes/no)” followed by a list of 14 substance categories with street name or prescription subtype examples, and an “Other” response. Polydrug use was operationalized as reports of more than two substances other than cannabis or alcohol.

**Table 1:** Baseline characteristics for youth at-risk for HIV by intervention arm and total analytic sample (N = 895)

	AMMI N = 313 N (%)	AMMI+coach N = 196 N (%)	AMMI+peer Support (PS) N = 205 N (%)	AMMI+PS+coach N = 181 N (%)	Total N = 895 N (%)
Age (years)					
Mean (SD)	21.1 (2.07)	20.9 (2.19)	21.0 (2.27)	21.2 (2.12)	21.0 (2.15)
Sex					
Female	27 (8.6%)	16 (8.2%)	11 (5.4%)	11 (6.1%)	65 (7.3%)
Male	286 (91.4%)	180 (91.8%)	194 (94.6%)	170 (93.9%)	830 (92.7%)
Gender					
Cisgender	249 (79.6%)	147 (75.0%)	173 (84.4%)	155 (85.6%)	724 (80.9%)
Gender diverse female	7 (2.2%)	3 (1.5%)	2 (1.0%)	5 (2.8%)	17 (1.9%)
Gender diverse male	18 (5.8%)	16 (8.2%)	6 (2.9%)	7 (3.9%)	47 (5.3%)
Transgender female	19 (6.1%)	17 (8.7%)	15 (7.3%)	8 (4.4%)	59 (6.6%)
Transgender male	20 (6.4%)	13 (6.6%)	9 (4.4%)	6 (3.3%)	48 (5.4%)
Sexual orientation					
Bisexual	78 (24.9%)	56 (28.6%)	51 (24.9%)	49 (27.1%)	234 (26.1%)
Gay	174 (55.6%)	102 (52.0%)	122 (59.5%)	108 (59.7%)	506 (56.5%)
Heterosexual	17 (5.4%)	11 (5.6%)	8 (3.9%)	5 (2.8%)	41 (4.6%)
Other non-heterosexual	3 (1.0%)	2 (1.0%)	0 (0%)	1 (0.6%)	6 (0.7%)
Pansexual	28 (8.9%)	18 (9.2%)	10 (4.9%)	11 (6.1%)	67 (7.5%)
Queer	12 (3.8%)	6 (3.1%)	13 (6.3%)	6 (3.3%)	37 (4.1%)
Missing	1 (0.3%)	1 (0.5%)	1 (0.5%)	1 (0.6%)	4 (0.4%)
Ethnicity					
White	60 (19.2%)	33 (16.8%)	47 (22.9%)	44 (24.3%)	184 (20.6%)
Asian/Pacific Islander/Native American	24 (7.7%)	12 (6.1%)	16 (7.8%)	10 (5.5%)	62 (6.9%)
Black/African American^+^	141 (45.0%)	77 (39.3%)	79 (38.5%)	65 (35.9%)	362 (40.4%)
Hispanic	80 (25.6%)	61 (31.1%)	58 (28.3%)	58 (32.0%)	257 (28.7%)
Other	8 (2.6%)	13 (6.6%)	5 (2.4%)	4 (2.2%)	30 (3.4%)
City					
Los Angeles	185 (59.1%)	120 (61.2%)	127 (62.0%)	114 (63.0%)	546 (61.0%)
New Orleans	128 (40.9%)	76 (38.8%)	78 (38.0%)	67 (37.0%)	349 (39.0%)
Education					
Below high school	55 (17.6%)	28 (14.3%)	30 (14.6%)	24 (13.3%)	137 (15.3%)
High school/equivalent	77 (24.6%)	47 (24.0%)	41 (20.0%)	41 (22.7%)	206 (23.0%)
Some higher education	144 (46.0%)	100 (51.0%)	94 (45.9%)	81 (44.8%)	419 (46.8%)
Completed higher education	34 (10.9%)	19 (9.7%)	35 (17.1%)	31 (17.1%)	119 (13.3%)
Missing	3 (1.0%)	2 (1.0%)	5 (2.4%)	4 (2.2%)	14 (1.6%)
Income greater than 2021 federal poverty level					
Yes	101 (32.3%)	79 (40.3%)	74 (36.1%)	54 (29.8%)	308 (34.4%)
No	212 (67.7%)	117 (59.7%)	131 (63.9%)	127 (70.2%)	587 (65.6%)
Insurance status					
Insured	235 (75.1%)	147 (75.0%)	166 (81.0%)	139 (76.8%)	687 (76.8%)
Uninsured/unsure	78 (24.9%)	49 (25.0%)	39 (19.0%)	42 (23.2%)	208 (23.2%)
What devices owned					
Cell phone	299 (95.5%)	189 (96.4%)	195 (95.1%)	172 (95.0%)	855 (95.5%)
No cell phone	14 (4.5%)	7 (3.6%)	10 (4.9%)	9 (5.0%)	40 (4.5%)
Amount of access to mobile devices*					
Not own device	26 (8.3%)	8 (4.1%)	11 (5.4%)	6 (3.3%)	51 (5.7%)
Own mobile device	258 (82.4%)	178 (90.8%)	184 (89.8%)	163 (90.1%)	783 (87.5%)
Own mobile device without minutes and/or without data	29 (9.3%)	10 (5.1%)	10 (4.9%)	12 (6.6%)	61 (6.8%)
Hazardous drinking					
Yes	126 (40.3%)	78 (39.8%)	93 (45.4%)	73 (40.3%)	370 (41.3%)
No	184 (58.8%)	117 (59.7%)	112 (54.6%)	106 (58.6%)	519 (58.0%)
Missing	3 (1.0%)	1 (0.5%)	0 (0%)	2 (1.1%)	6 (0.7%)
Marijuana use (recent)					
Yes	231 (73.8%)	142 (72.4%)	140 (68.3%)	135 (74.6%)	648 (72.4%)
No	82 (26.2%)	53 (27.0%)	65 (31.7%)	44 (24.3%)	244 (27.3%)
Missing	0 (0%)	1 (0.5%)	0 (0%)	2 (1.1%)	3 (0.3%)
Marijuana use (lifetime)					
Yes	271 (86.6%)	163 (83.2%)	170 (82.9%)	158 (87.3%)	762 (85.1%)
No	42 (13.4%)	32 (16.3%)	35 (17.1%)	21 (11.6%)	130 (14.5%)
Missing	0 (0%)	1 (0.5%)	0 (0%)	2 (1.1%)	3 (0.3%)
Polydrug use (recent)					
Yes	118 (37.7%)	73 (37.2%)	78 (38.0%)	74 (40.9%)	343 (38.3%)
No	195 (62.3%)	123 (62.8%)	127 (62.0%)	105 (58.0%)	550 (61.5%)
Missing	0 (0%)	0 (0%)	0 (0%)	2 (1.1%)	2 (0.2%)
Polydrug use (lifetime)					
Yes	181 (57.8%)	112 (57.1%)	115 (56.1%)	113 (62.4%)	521 (58.2%)
No	132 (42.2%)	84 (42.9%)	90 (43.9%)	66 (36.5%)	372 (41.6%)
Missing	0 (0%)	0 (0%)	0 (0%)	2 (1.1%)	2 (0.2%)
Substance abuse treatment program (lifetime)					
Yes	59 (18.8%)	31 (15.8%)	27 (13.2%)	24 (13.3%)	141 (15.8%)
No	252 (80.5%)	165 (84.2%)	178 (86.8%)	157 (86.7%)	752 (84.0%)
Missing	2 (0.6%)	0 (0%)	0 (0%)	0 (0%)	2 (0.2%)
Homelessness (lifetime)^a^					
Yes	140 (44.7%)	57 (29.1%)	58 (28.3%)	62 (34.3%)	317 (35.4%)
No	173 (55.3%)	139 (70.9%)	147 (71.7%)	119 (65.7%)	578 (64.6%)
Incarceration (lifetime)^a^					
Yes	62 (19.8%)	23 (11.7%)	26 (12.7%)	30 (16.6%)	141 (15.8%)
No	249 (79.6%)	173 (88.3%)	178 (86.8%)	150 (82.9%)	750 (83.8%)
Missing	2 (0.6%)	0 (0%)	1 (0.5%)	1 (0.6%)	4 (0.4%)
Sex exchange (lifetime)^a^					
Yes	93 (29.7%)	40 (20.4%)	43 (21.0%)	41 (22.7%)	217 (24.2%)
No	219 (70.0%)	153 (78.1%)	162 (79.0%)	139 (76.8%)	673 (75.2%)
Missing	1 (0.3%)	3 (1.5%)	0 (0%)	1 (0.6%)	5 (0.6%)
Hospitalization for mental health (lifetime)^a^					
Yes	94 (30.0%)	45 (23.0%)	36 (17.6%)	31 (17.1%)	206 (23.0%)
No	219 (70.0%)	151 (77.0%)	169 (82.4%)	150 (82.9%)	689 (77.0%)

^a^Chi-squared tests of independence for imbalances at baseline between arms p < 0.05

^+^Includes youth specifying both Black-non-Hispanic and Black-Hispanic

### Data Analyses

We present results for GLMM models fit to the analytic sample. Results were similar for GLMM and GEE, analytic and baselined samples (n = 895 and 1037, respectively), and models with and without adjustment covariates. We favored GLMM because it provided an additional missing data adjustment. We present results with and without covariate adjustments with a focus on the latter given focus on intervention effects rather than factors associated with baseline services use.

We used chi-square tests to compare the sociodemographic characteristics and risk histories of youth in each intervention condition and t-tests to compare continuous variables. We then used an intent-to-treat logistic regression analysis to compare any support service utilization across the four intervention conditions. The model included covariates for intervention condition random assignment, time from baseline measurement, and intervention condition by time interactions to indicate intervention effects. The model included a linear time trend based on plots of the frequencies of each service used over time. We accounted for correlations between repeated outcome measurements by fitting a generalized linear mixed effects model (GLMM) with a random intercept to the data using a penalized quasi-likelihood (PQL) method through the MASS package in R [45]. Finally, to investigate the possibility of differential intervention effects between the two cities given baseline differences in service use, we conducted a sensitivity analysis fitting a model that included a three-way interaction between the intervention arm, time (visit), and city using the same methods described above.

The analysis of the other healthcare utilization variables proceeded similarly. We fit GLMMs for each of the six outcomes with intervention condition, time from baseline measurement, and intervention condition by time interactions. We assumed Poisson or binomial distributions for count and binary outcomes, respectively. An additional quadratic time term and a negative binomial outcome was used for the mental health support group and HIV prevention program outcomes, based on preliminary exploration of the data. Because there were six outcome variables, we performed a Bonferroni correction by setting alpha equal to 0.05/6. Non-quadratic time models were fit using PQL and the quadratic time models were fit using Bayesian methods with the brms package in R [46].

Analyses assume that randomization was successful in approximately balancing participant characteristics across conditions. As a sensitivity analysis of this assumption, we fit GLMM models including covariates that differed significantly between intervention conditions (p < 0.05 in Table 1), are hypothesized to be correlated with services usage, associated with lower retention (see Online Resource 2), and randomization stratification variables (race/ethnicity and sexual orientation). Analyses also controlled for enrollment date since participants were more likely to be randomized to AMMI earlier in recruitment, and COVID-19 onset of March 17, 2020 when L.A. and New Orleans began stay at home orders that may have impacted study retention and services availability. These adjusted analyses used complete case analysis. Given the modest levels of missing covariate data shown in Table 1, each analysis had slightly lower sample sizes than 895 cases in the main analytic sample, ranging from 873 to 886 depending on outcome variable (noted in the respective Tables).

## Results

The sociodemographic characteristics and risk histories of SGMY in the four conditions and overall are summarized on Table 1. Youth were about 21 years of age, ranging from 16 to 24. Most were male (93%), cis-gender (81%) and about half (57%) self-identified as gay, 26% as bisexual and about 19% as transgender/gender-diverse. Most youth were Black (40%, including 64 [7%] also reporting other races or ethnicities) or Latino (29%). There were about 6% Asian/Pacific Islander, and 4% other ethnicities. More youth (61%) were recruited in Los Angeles than in New Orleans (39%). About 38% of youth had less than a high school education or a diploma, almost half (47%) had some post-high school education and 13% had graduated from college. Only a minority were above the poverty line (34%), although most had health insurance (76%). Lifetime homelessness was reported by 35%, mental health hospitalizations by 23%, and incarceration by 16%.

Hazardous drinking was reported by 41%, with 72% recently using marijuana and more than 85% reporting lifetime marijuana use. Lifetime polysubstance use (excluding cannabis) was reported by 58%, with 38% reporting use in past 4 months. About 16% had attended substance use treatment programs. Almost one in four participants (24%) reported having exchanged sex for drugs or money in their lifetime.

**Fig. 2:**
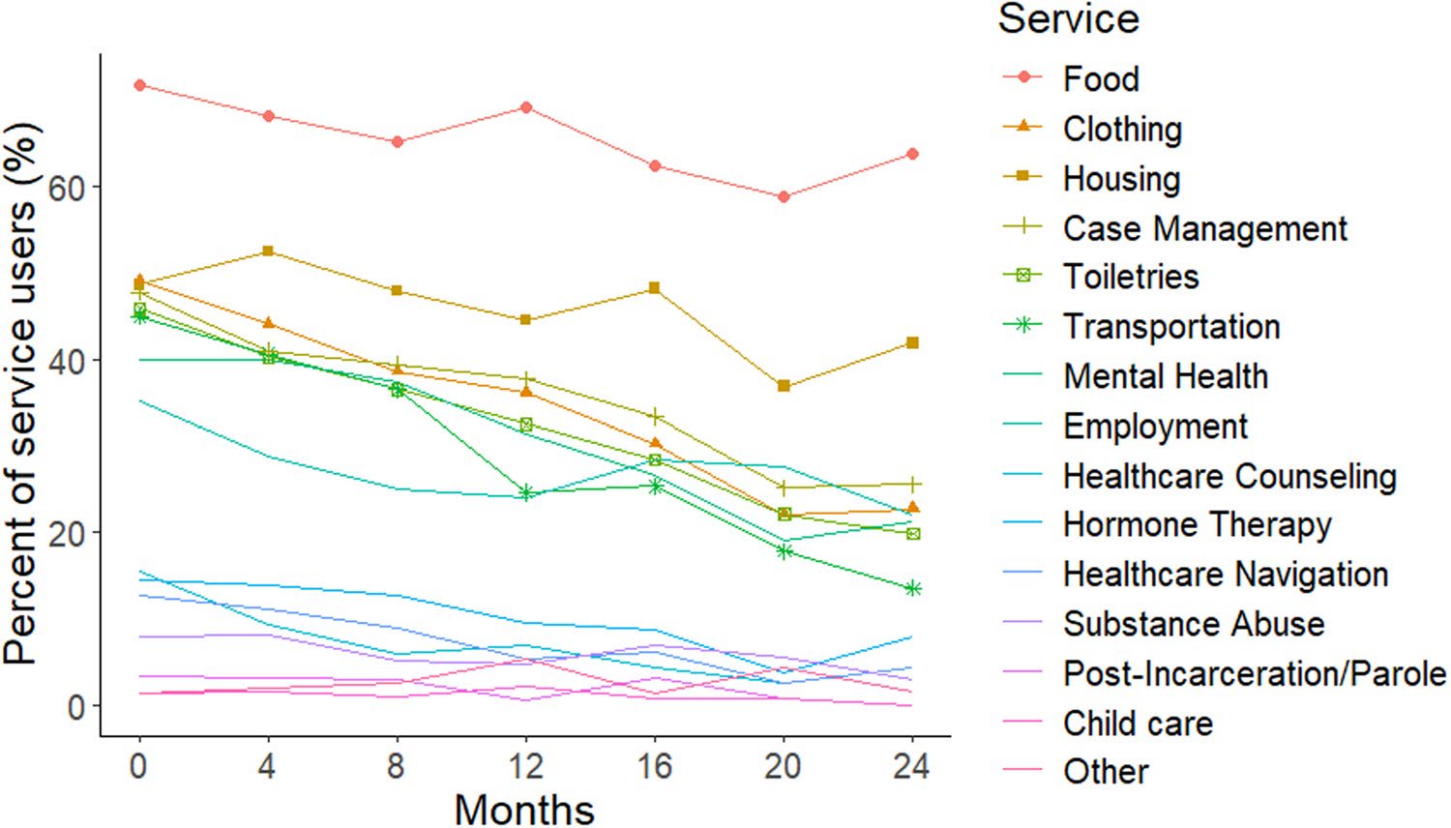
Percent of youth among service users using each type of service over time

Support service use was reported by 57.5% (n = 515) of participants at one or more study assessments, with 13% (n = 116) at all assessments. Overall, support services use declined over time across the service types. Table 2 summarizes the percentage of youth over time who used any service at each time point (40% baseline – 15.8% at 24 months) as well as the Mean (SD) number of services used by those with at least one service are (M = 4.4, SD = 3.1 at baseline to M = 2.5, SD = 2.2 at 24 months). Figure 2 summarizes the frequency of the types of services utilized at each follow-up point, demonstrating that food, clothing, and housing were used most frequently at each timepoint.

**Table 2:** Summary of the percentage of youth-at-risk for HIV who received at least one service in the last four months, the mean number and standard deviation (SD) of services used by all youth at each assessment at four-month intervals over 24 months

Visit	Percent using services	Number of people who completed follow-up assessment	Mean (SD) number of services used among users
0–baseline	40%	895	4.40 (3.06)
1–4-months	32.4%	805	4.04 (2.94)
2–8-months	30.9%	764	3.65 (2.75)
3–12-months	26.9%	709	3.34 (2.41)
4–16-months	24.3%	666	3.14 (2.75)
5–20-months	25.8%	632	2.49 (2.31)
6–24-months	22.6%	625	2.51 (2.24)

Service users are defined as participants who reported at least one service in the past 4 months of their visit

The “Number of people who completed follow-up assessment” column is used as the denominator to calculate the “Percent using services” column

### Intervention Effects

**Fig. 3:**
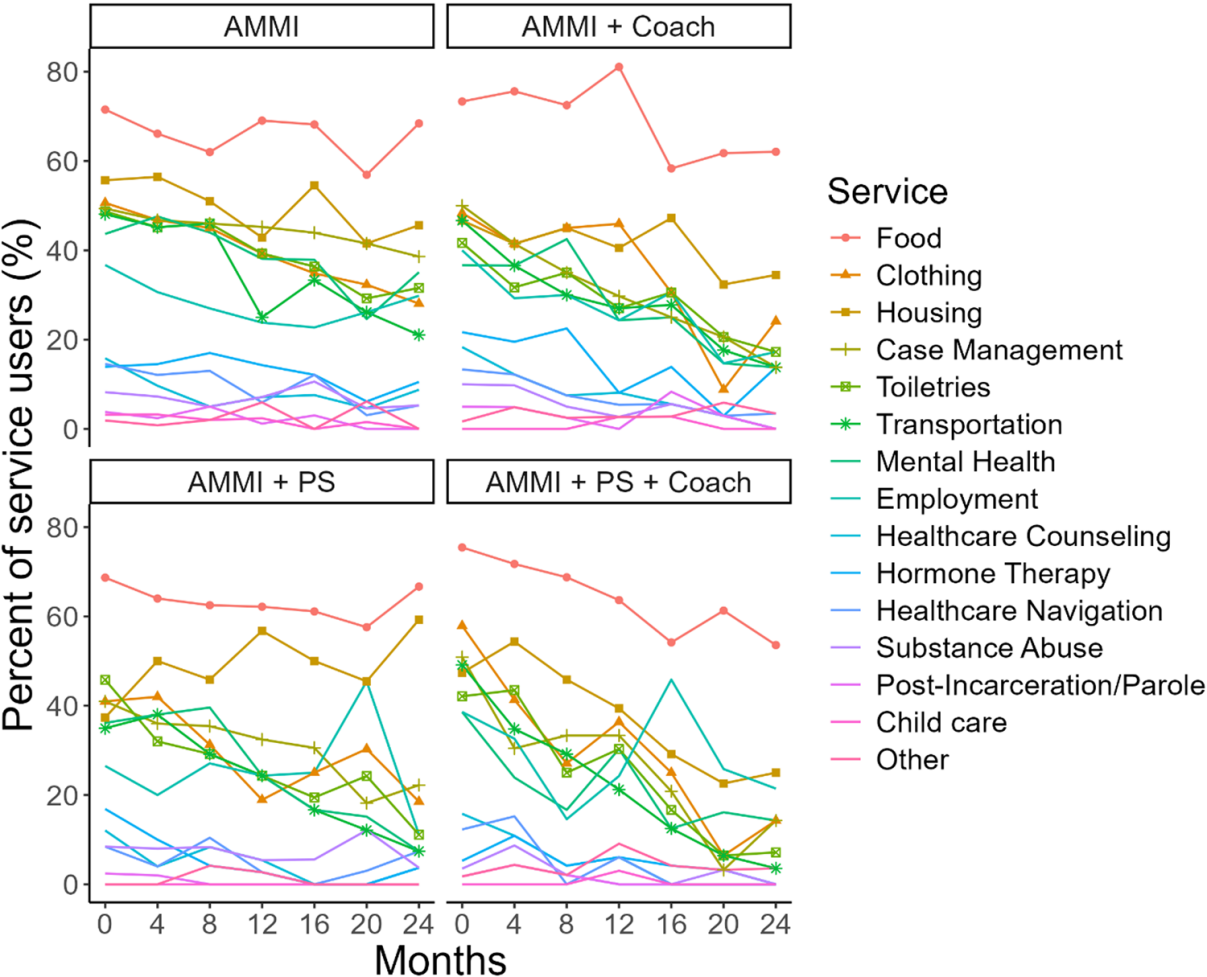
Percent of service users who used each category of services over time for each intervention condition: AMMI, AMMI+coaching, AMMI+peer support (PS), AMMI+PS+coaching

We focus detailed presentation on support services results, followed by other services use variables given their lack of statistically significant intervention arm differences over time. Figure 3 shows the frequency of each type of support service utilized by youth in each intervention condition over time. Across arms (Figs. 2 and 3), food and housing services were the most frequently used. Substance use treatment, post-incarceration services and childcare were the least used services, reflecting more specialized needs. These patterns were relatively consistent over time, with the frequency of each type of service decreasing across time.

**Fig. 4:**
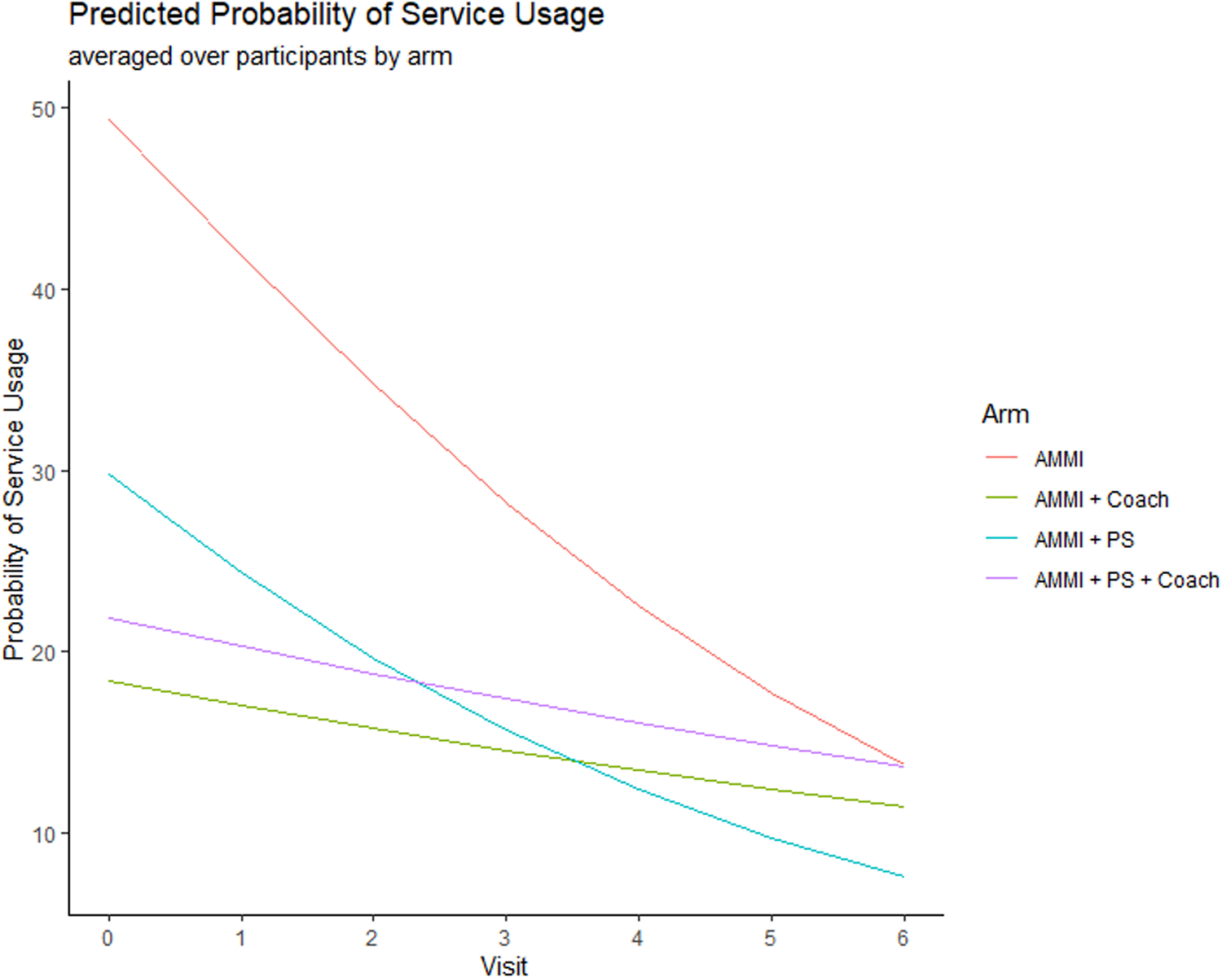
Averaged predicted probability of using at least one service across intervention conditions over 24 months from results of the Generalized Linear Mixed Model (GLMM) regressions from Table 3

Figure 4 plots the overall probability of service utilization over time for each intervention condition predicted by GLMM. Significantly more services are reported at the baseline assessment by youth in the AMMI only condition compared to the other three conditions, as can also be seen from the regression coefficients in Table 3 with all the confidence intervals (CI) for the odds ratios of the baseline condition values below one compared to AMMI only reference group. Due to protocol changes noted above, more participants were enrolled in the AMMI-only arm earlier in the study and were more likely to be recruited from homeless services sites and less likely to have been recruited via social media outreach, therefore, we also estimated contrasts (comparisons) to the AMMI+PS arm that had the same recruitment timelines as the two coaching arms.

**Table 3:** Ancillary support services use results from generalized linear mixed model (GLMM) with random intercept for each participant comparing intervention condition slopes over time to automated messaging and monitoring intervention (AMMI) only (N = 895)

Variable	Odds ratio estimate	95% confidence interval	99.17% confidence interval^
Intercept	0.97	0.71–1.34	0.64–1.49
AMMI+coaching (C)	0.23	0.14–0.39^a^	0.11–0.47^a^
AMMI+peer support (PS)	0.43	0.26–0.72^a^	0.22–0.86^a^
AMMI+PS+C	0.29	0.17–0.49^a^	0.14–0.59^a^
Visit	0.74	0.7–0.78^a^	0.69–0.80^a^
Visit × AMMI+C	1.23	1.12–1.35^a^	1.09–1.40^a^
Visit × AMMI+PS	1.03	0.94–1.12	0.91–1.16
Visit × AMMI+PS+C	1.23	1.12–1.35^a^	1.09–1.39^a^

^Bonferroni corrected 99.17% confidence intervals (based on α = 0.05 / 6 = 0.0083) for six outcomes

^a^Indicates that the confidence interval for odds ratio does not contain 1

Across conditions support service usage decreased over time in an approximately linear fashion as we can see from Figs. 2, 3, and 4. Table 3 shows regression coefficient point estimates, 95% and 99.17% confidence intervals for the intervention effects model. The OR for visit is less than 1, also indicating that there is an overall decrease in service usage over time for the AMMI condition (OR 0.74, 95% CI 0.70–0.78). The interaction effects between the intervention conditions and time represent trajectory differences compared to the AMMI condition. The OR estimate for AMMI+C by time interaction is greater than one (OR 1.23, 95% CI 1.12–1.35; 99.17% CI 1.09–1.40), which shows that the rate of service usage does not decline as fast as it does in the AMMI condition. The point estimate and confidence interval for the AMMI+PS+C condition by time interactions are practically identical (OR 1.23, 95% CI 1.12–1.35; 99.17% CI 1.09–1.39). The PS condition by time interaction includes one and is insignificant, and therefore, similar to AMMI alone (OR 1.03, 95% CI 0.94–1.12; 99.17% CI 0.91–1.16). Comparing the two coaching arms to AMMI+PS finds similar results with practically identical ORs and CIs; AMMI+C (OR 1.20, 95% CI 1.08–1.33; 99.17% CI 1.04–1.38) and AMMI+PS+C (OR 1.20, 95% CI 1.08–1.33; 99.17% CI 1.04–1.37).

Analyses did not indicate differential follow-up retention by study arm (Supplemental Tables 1, 2, 3b & Figs. 3, 4) but baseline factors associated with loss to follow-up included: lower education, income, PrEP use, and support services use; experiencing lifetime homelessness, incarceration, mental health hospitalization, and interpersonal violence; not owning their own cell phone; and recent cannabis use.

Table 4 shows regression coefficient point estimates and 95% and 99.17% confidence intervals for the support services outcome model including additional adjustment covariates. The main conclusions remain the same. Conditions including the coaching intervention have similar time trends and are significantly different from the AMMI arm and the AMMI+PS arm. That is, the rate of service usage decline is slower in the conditions involving the AMMI+C or AMMI+PS+C intervention. We also observe several variables associated with higher support services use at baseline: transgender or gender diverse (vs. cis-gender), bisexual or other (vs. gay), Los Angeles (vs. New Orleans); lifetime homelessness, sex exchange, and hospitalized for mental health; and earlier enrollment date. The GLMM results for intervention effects are similar, with or without these covariates included.

**Table 4:** Ancillary support services use results of general linear mixed model (GLMM) regression model comparing intervention condition slopes over time to automated messaging and monitoring intervention (AMMI) only for, controlling for baseline covariates (N = 878)

Variable	Odds ratio estimate	95% confidence interval	99.17% confidence interval^
Intercept	0.09	0.02–0.42*	0.01–0.72*
AMMI+coaching (C)	0.57	0.35–0.92*	0.30–1.09
AMMI+peer support (PS)	1.14	0.72–1.80	0.62–2.11
AMMI+PS+C	0.66	0.41–1.05	0.35–1.24
Visit	0.67	0.63–0.71*	0.61–0.73*
Visit × AMMI+C	1.19	1.08–1.31*	1.04–1.36*
Visit × AMMI+PS	1.01	0.92–1.11	0.89–1.15
Visit × AMMI+PS+C	1.23	1.12–1.36*	1.08–1.40*
Age	1.05	0.98–1.12	0.96–1.15
Gender [ref group = cis-gender]			
Trans/Gender Diverse	2.60	1.72–3.93*	1.49–4.53*
Race [ref group = white, non-hispanic]**			
Black	1.08	0.72–1.62	0.62–1.86
Latino	0.96	0.64–1.44	0.55–1.66
Other	0.87	0.50–1.51	0.41–1.83
Sexual orientation [ref group = gay]			
Bisexual	2.10	1.51–2.93*	1.35–3.29*
Other	1.61	1.03–2.52*	0.88–2.94
City [ref group = Los Angeles]			
New Orleans	0.41	0.29–0.58*	0.25–0.65*
Has insurance	1.23	1.00–1.52	0.92–1.64
Income above federal poverty level	0.74	0.54–1.01	0.49–1.13
Device access [ref group = own mobile device]			
Not own device	1.85	0.98–3.50	0.79–4.36
Own mobile device, but without minutes and/or without data	1.11	0.64–1.94	0.52–2.35
Homelessness (lifetime)	7.72	5.45–10.93*	4.84–12.33*
Incarceration (lifetime)	1.50	0.99–2.27	0.86–2.62
Sex exchange (lifetime)	1.47	1.05–2.04*	0.94–2.29
Hospitalized for mental health (lifeitme)	1.47	1.04–2.09*	0.92–2.36
After COVID onset (March 17, 2020)	1.97	1.53–2.53*	1.40–2.75*
Enrollment date+	0.77	0.71–0.83*	0.69–0.86*

Sample size reduced due to missing data in some covariates for complete case analysis

^Bonferroni corrected 99.17% confidence intervals

^+^Entered at midpoint enrollment time and scaled by 100 days

^**^Other races include: Asian, Hawaiian/ Pacific Islander, Native American, Alaskan Native, and other

Sensitivity analysis examining potential for differential intervention effects by city also did not indicate such effects (Supplemental Table 4). Although there were statistically significant baseline differences in support services use across cities, none of the three-way interactions between arms, time (visit), and city were statistically significant. Furthermore, the effects of the two coaching intervention arms on support service usage over time remained statistically significant with similar ORs and confidence intervals to results reported above without the city interaction.

**Fig. 5:**
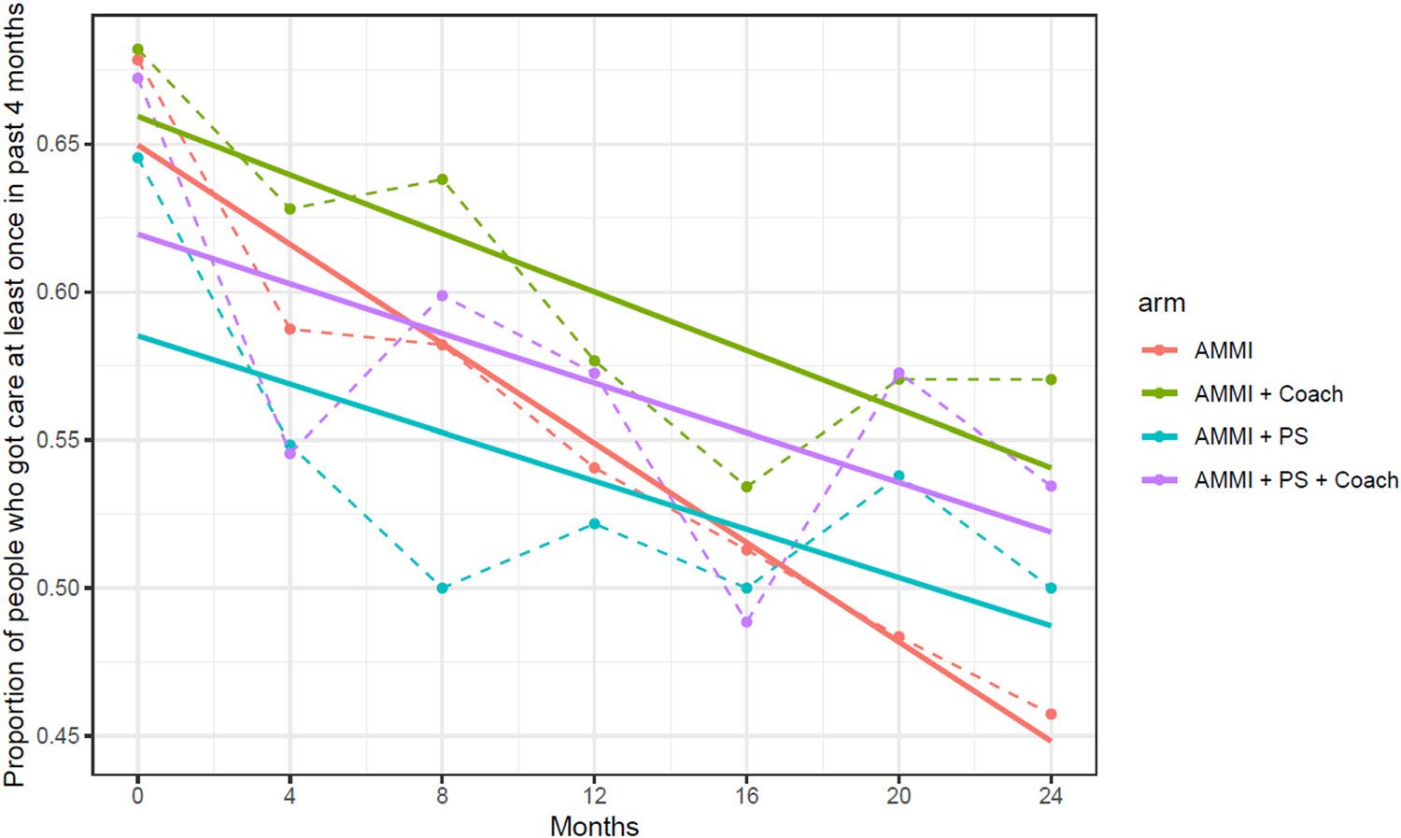
Received care from doctors office or clinic over 24 months across intervention conditions

There were intervention effect trends for having received care from a doctor’s office or clinic in the past four months for all arms in comparison to AMMI-Only, but results were not robust with covariate and Bonferroni adjustments, nor for comparisons to AMMI+PS (Fig. 5 and Table 5). Figure 5 shows declining use over time and similar slopes for arms except AMMI-Only. Table 5 shows unadjusted and adjusted model results, with both showing intervention effect (Visit x Arm) estimates losing statistical significance in Bonferroni adjusted CIs; AMMI+C (OR 1.07, 95% CI 0.98–1.16; 99.17% CI 0.96–1.19) and AMMI+PS+C (OR 1.09, 95% CI 1.01–1.19; 99.17% CI 0.98–1.22). Results were similar for AMMI+PS vs. AMMI-Only (OR 1.09, 95% CI 1.00–1.18; 99.17% CI 0.98–1.21) and similar to results for the two coaching arms shown above, thus indicating no differences between the coaching and peer support arms.

**Table 5:** Estimated intervention effects for receiving care from doctor’s office or clinic in the past 4 months without covariate adjustment (N = 895), and with covariate adjustment (N = 886)

				Adjusted model with covariates (N = 886)		
Variable	Odds ratio estimate	95% confidence interval	99.17% confidence interval^	Odds ratio estimate	95% confidence interval	99.17% confidence interval^
Intercept	2.15	1.74–2.67^a^	1.62–2.87^a^	1.10	0.35–3.45	0.24–5.12
AMMI+coach	1.01	0.72–1.43	0.64–1.62	1.00	0.69–1.43	0.61–1.63
AMMI+PS	0.73	0.52–1.03	0.47–1.16	0.72	0.51–1.01	0.45–1.15
AMMI+PS+coach	0.83	0.58–1.17	0.52–1.32	0.79	0.55–1.14	0.49–1.29
Visit	0.83	0.79–0.87^a^	0.78–0.89^a^	0.81	0.77–0.86^a^	0.75–0.88^a^
Visit x AMMI+coach	1.08	1.00–1.17	0.97–1.2	1.07	0.98–1.16	0.96–1.19
Visit x armAMMI+PS	1.09	1.01–1.18^a^	0.99–1.21	1.09	1.00–1.18^a^	0.98–1.21
Visit x armAMMI+PS+Coach	1.09	1.01–1.18^a^	0.98–1.22	1.09	1.01–1.19^a^	0.98–1.22

*PS* peer support

^a^confidence interval does not include 1

^Bonferroni corrected confidence intervals based on alpha = .05 / 6 = .0083 for six outcomes

Finally, we did not observe intervention arm differences over time for the other outcome variables (Supplemental Fig. 5, 6, 7, 8 and Tables 5, 6, 7, 8). Having a regular healthcare provider was stable or trended toward slight increase across arms over time. There were declining reports across arms of mental health specialist care, mental health self-help support groups, and HIV prevention program participation.

## Discussion

Most studies on service utilization examine improvement in a specific domain. For example, youth at risk for HIV are evaluated for how they access and adhere to HIV prevention services and behaviors; homeless youth are evaluated for accessing shelter and housing services. In contrast, we focus on youth’s utilization of services in multiple domains given our intervention’s recognition of the importance of supporting youth to address their priorities and competing needs in conjunction with, or wholistic support for, HIV prevention [22, 47]. HIV prevention is difficult to prioritize when facing more proximal challenges of daily living (e.g., housing, food, and economic insecurities), mental health, social connection, and other health priorities [47–51]. Rather than focusing on HIV services only in health care settings, it is critical to support utilization of a range of support services.

Overall, across conditions over time, participants reported decreased use of support, healthcare, mental health, and HIV prevention program services, and stable reports of having a regular healthcare provider. However, the decreases in support services utilization were lower or flatter in the two coaching study arms compared to both the AMMI-only and AMMI+PS conditions. There are several possible explanations, including methodological limitations. First, outcomes are based on self-reports, so results should be interpreted with caution given potential reporting biases such as training effects to avoid question burden, and social desirability for higher or lower services reporting potentially influenced by higher contact and contact attempts in the coaching interventions, although similar contact attempts were made for the peer support intervention. Other limitations may include regression to the mean and loss to follow-up of more marginalized participants with higher services needs or who were less likely to utilize services. However, loss to follow-up did not differ significantly across study arms and the statistical analyses included missing data and covariate adjustments. Limitations aside, needs may have been met and challenges addressed generally over time at recruitment or referral sites, and possibly supported by study procedures as participants received referral information from study interviewers and AMMI [40]. Service utilization may also have declined due to difficulties accessing service systems such as missing appointments and rescheduling, or follow-through or persistence in navigating eligibility and availability.

The potential intervention effects are consistent with coaches’ roles and training to motivate and support youth through warm hand-off linkages (rather than passive referrals), goal setting with youth for follow-through on linkages, consistent follow ups on goal progress, and problem-solving difficulties. Thus, coaching functioned partly as peer navigation, an evidence-based intervention strategy that improves engagement in health care among hardly reached populations [48]. Evidence-based cognitive-behavioral skills training such as problem solving, assertive communication, and rehearsal or role-play with coaches may have supported effective communication with services providers for some participants. The intervention modalities of reminders (texting), peer support, navigation and coaching are also highly similar to the recent recommendations from metanalyses of HIV care continuum interventions conducted by the CDC Synthesis Team [52].

We should also acknowledge the limitations of digital technology mediated interventions for reach and effectiveness with the most marginalized youth who may have no or intermittent phone access, and their potential to perpetuate health disparities. Our disruptive innovations approach using simpler and lower cost technologies instead of smartphone apps to be accessible to more youth is consistent with public health prevention approaches rather than highly specialized intervention for highest need clients [28, 29]. Although we did not encounter significant cell phone related barriers at screening and enrollment, we did encounter difficulties for some participants over time with maintaining wireless service consistently or loss to contact. Our coaching and interviewer teams found that wireless service tended to be reactivated at the beginning of a month when General Assistance or General Relief (GA/GR) Program payments were issued by local counties, although more available in Los Angeles than New Orleans. We previously reported factors associated with weekly text-message survey response rate patterns, with over 2/3 responding at least once and 45% response rates on average over time; about 41% were consistent responders, 34% non-responders (or inconsistent), and 24% had wireless service related non-responses [53]. Lower response patterns, with and without wireless service issues, were generally predicted by being younger, Black and Latinx, homeless in lifetime, incarcerated in lifetime, and having higher recent support services utilization, which are similar to the factors associated with study retention reported above. The primary outcome paper previously reported participation rates of about three quarters receiving text-messages, over half participating in coaching, but only about a quarter participating in peer support, indicating potential technology barrier for a mobile-web app requiring a login ID and password to access [21]. We are currently conducting analyses on predictors of intervention participation and study retention to identify characteristics of SGMY who might benefit from additional strategies to enhance participation or might need or prefer different intervention strategies. Yet, the main result of this paper is that coaching resulted in greater reports of services engagement and was also offered in-person at community partner sites where participants could be linked to more specialized services, such as housing programs, case management, and mental health specialists.

Finally, it is unclear whether utilizing support services is related to better long-term outcomes. There may also be concerns about costs associated with services utilization to healthcare providers, social services agencies, and insurers or payers, particularly if there is not a resulting improvement in health or functional outcomes. Yet, the goal of support services is to improve daily living, physical and mental health, and quality of life. In the long run, these benefits may also avert infections and lower overall lifetime costs at other levels. Future analyses will model intervention and societal costs for cost-effectiveness analyses.

## Supplementary Information

Below is the link to the electronic supplementary material.

Supplementary file1 (DOCX 1627 KB)

## Data Availability

Data will be made available upon request and will also be available on the NICHD DASH data repository.
