# Supplementary material for: Strategies to Facilitate Service Utilization Among Youth at Risk for HIV: A Randomized Controlled Trial (ATN 149)
Source: AIDS Behav. 2024 Nov 28;29(2):626–41. doi: 10.1007/s10461-024-04545-2 (PMC11813953; doi:10.1007/s10461-024-04545-2)
Supplement: Supplementary file 1 — Supplementary file1 (DOCX 1627 KB) [file 10461_2024_4545_MOESM1_ESM.docx]

Supplementary Materials for “Strategies to Facilitate Service Utilization among Youth at Risk for HIV:

A Randomized Controlled Trial (ATN 149). *AIDS & Behavior.*

Supplementary Figure 1: Percentage of evidence-based practice elements used in coaching sessions as reported by coaches in the mobile-web case management system.


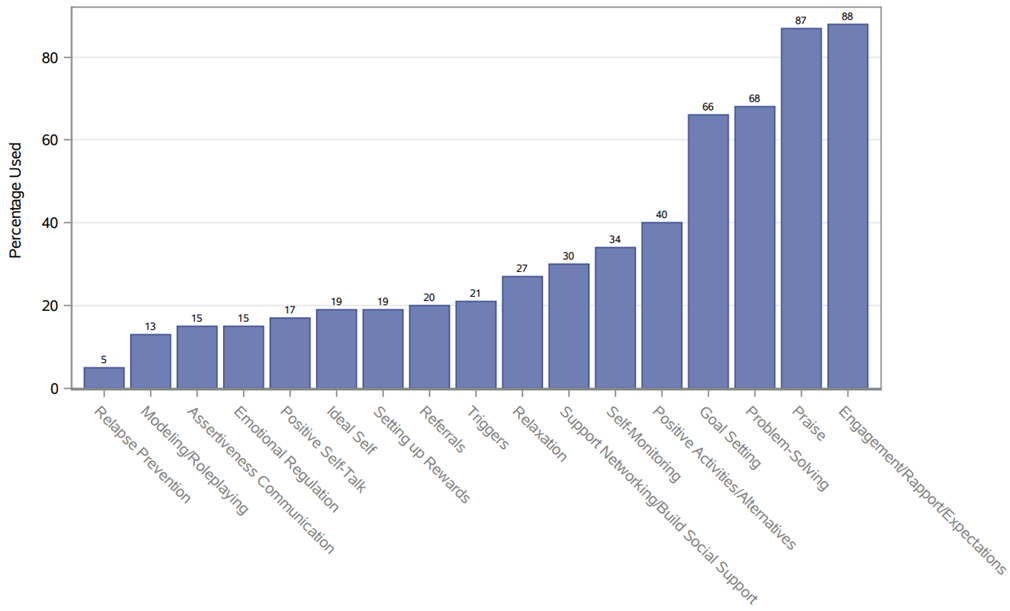


Supplementary Figure 2: Percentage of content areas addressed in coaching sessions as reported by coaches in the mobile-web case management system


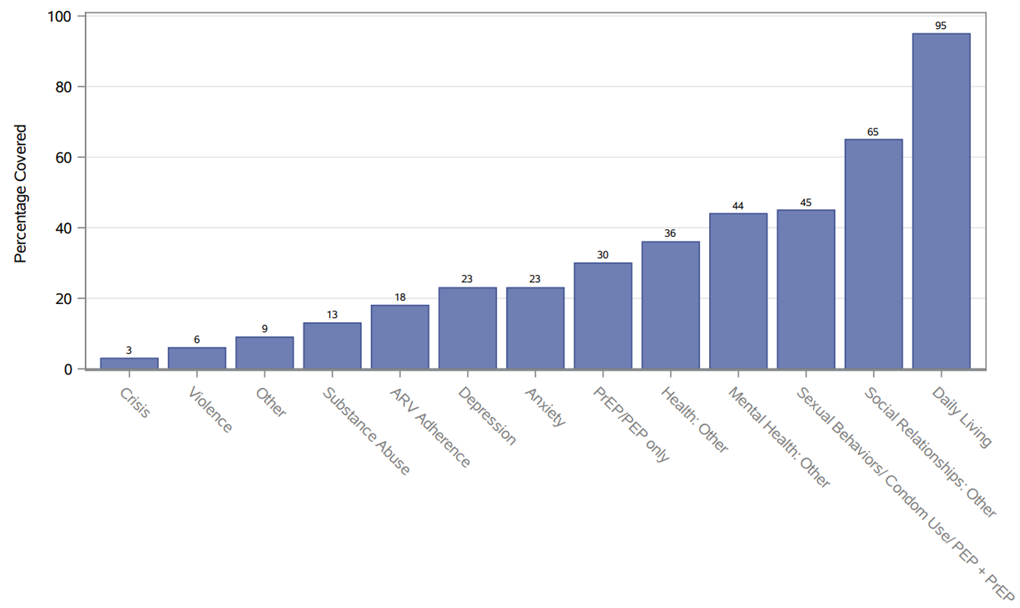


**Online Resource 2: Study Visit Follow-Up Retention Analyses**

**Summary**

We investigate follow-up retention by study arm and differences in participant characteristics across different degrees of loss to follow up analytic sample (N = 895) that excludes baseline only participants in figures and tables below. Key summary findings are as follows:

**Loss to follow-up difference by intervention arms is minimal.**

Supplemental Tables 1 & 2 and Figures 3 & 4 demonstrate that the rate of loss to follow up over times appears fairly even across arms, and that the majority of participants had all 6 study visits. If total number of follow-up visits is used as a metric to evaluate loss to follow up, then we again see that there is no significant difference between arms in mean number of total follow-up visits that each participant had.

**Baseline participant characteristic differences between differing loss to follow up.**

Supplemental Tables 3a & 3b show baseline characteristics of participants comparing those with only 1 follow-up visit vs. participants with 2 or more follow-up visits (Table 3a) and with and without follow-up visits in their second year of the study (Table 3b). In general, participants with less follow-up assessments were more likely to:

- be less educated
- have a lower income
- use cannabis in past 4 months
- have been hospitalized for mental health before (lifetime)
- have been homeless before (lifetime)
- have been incarcerated before (lifetime)
- have experienced interpersonal violence in their lifetime
- not have used PrEP (lifetime or past 4 months)
- not have used services in past 4 months
- not own their own cell phone

Supplemental Table 1: Numbers and Percentages of Study Visits Completed by Arm (N = 895)

| # of Visits | AMMI | AMMI + Coach | AMMI + PS | AMMI + PS + Coach |
| --- | --- | --- | --- | --- |
| 1 | 24 (7.67%) | 12 (6.12%) | 10 (4.88%) | 11 (6.08%) |
| 2 | 27 (8.63%) | 11 (5.61%) | 13 (6.34%) | 19 (10.5%) |
| 3 | 19 (6.07%) | 17 (8.67%) | 27 (13.17%) | 16 (8.84%) |
| 4 | 32 (10.22%) | 30 (15.31%) | 13 (6.34%) | 20 (11.05%) |
| 5 | 58 (18.53%) | 39 (19.9%) | 47 (22.93%) | 33 (18.23%) |
| 6 | 153 (48.88%) | 87 (44.39%) | 95 (46.34%) | 82 (45.3%) |

Notes: PS = Peer Support. Chi-squared test for independence indicate no significant association between number of follow-up visits and intervention arm (𝜒2=22.169, df=15, p=0.1034). Percentages are based on number of participants in each arm (column percentages), for example, the AMMI+Coaching arm had 12 participants with only one follow-up visit; these 12 participants make up 6.12% of the total number of participants in the AMMI+Coaching group. Supplemental Figure 6 below shows number of study visits across all study arms.

**Supplemental Figure 3. Number of Follow-Up Study Visits Completed (n=895).**


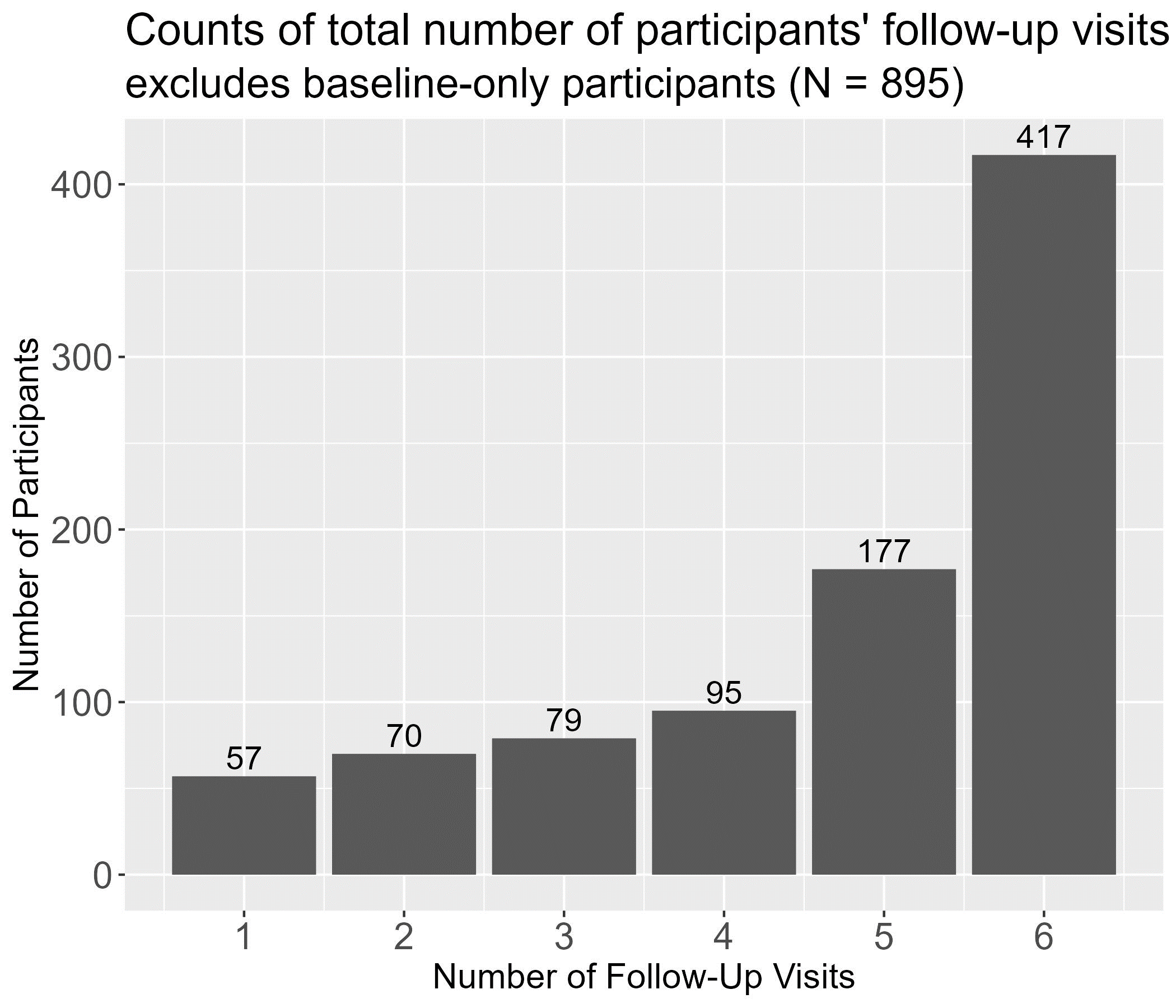


Supplemental Table 6 and Supplemental Figure 7 show further elaborates that there is little difference in retention across arms.

**Supplemental Table 2. Retention of Participants in Each Study Intervention Arm Over Time**

|  | **Months Since Baseline** | | | | | | |
| --- | --- | --- | --- | --- | --- | --- | --- |
| **Arm** | **0** | **4** | **8** | **12** | **16** | **20** | **24** |
| AMMI | 313 (100%) | 288 (92%) | 269 (85.9%) | 246 (78.6%) | 232 (74.1%) | 213 (68.1%) | 223 (71.2%) |
| AMMI + Coach | 196 (100%) | 165 (84.2%) | 164 (83.7%) | 163 (83.2%) | 146 (74.5%) | 149 (76%) | 135 (68.9%) |
| AMMI + Peer Support | 205 (100%) | 187 (91.2%) | 174 (84.9%) | 162 (79%) | 156 (76.1%) | 145 (70.7%) | 150 (73.2%) |
| AMMI + Peer Support + Coach | 181 (100%) | 165 (91.2%) | 157 (86.7%) | 138 (76.2%) | 132 (72.9%) | 125 (69.1%) | 117 (64.6%) |
| Total | 895 (100%) | 805 (89.9%) | 764 (85.4%) | 709 (79.2%) | 666 (74.4%) | 632 (70.6%) | 625 (69.8%) |

**Supplemental Figure 4. Retention of Participants in Each Intervention Arm Over Time**


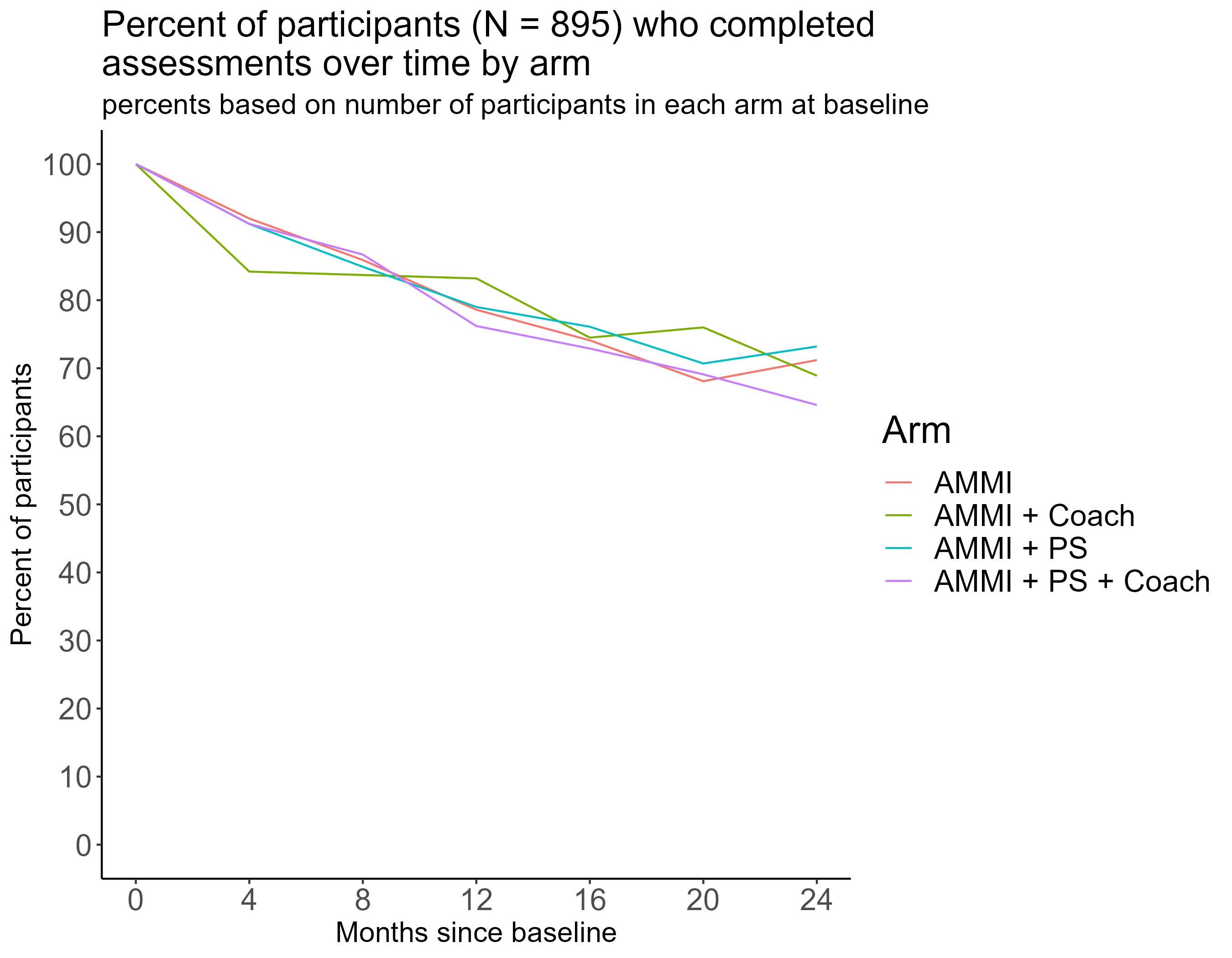


Supplemental Table 3a. Comparing characteristics of participants with only 1 follow-up visit vs. participants with 2 or more follow-up visits.

|  | 0-1 follow-up visits | 2 or more follow-up visits | Overall | Chi-Square Test / T-Test Statistic | P-value |
| --- | --- | --- | --- | --- | --- |
|  | (N=57) | (N=838) | (N=895) |  |  |
| Arm |  |  |  |  |  |
| AMMI | 24 (42.1%) | 289 (34.5%) | 313 (35.0%) | 1.70 | 0.638 |
| AMMI + Coach | 12 (21.1%) | 184 (22.0%) | 196 (21.9%) |  |  |
| AMMI + PS | 10 (17.5%) | 195 (23.3%) | 205 (22.9%) |  |  |
| AMMI + PS + Coach | 11 (19.3%) | 170 (20.3%) | 181 (20.2%) |  |  |
| Age (years) |  |  |  |  |  |
| Mean (SD) | 20.9 (2.05) | 21.0 (2.16) | 21.0 (2.15) | -0.49 | 0.627 |
| Median [Min, Max] | 21.0 [16.0, 24.0] | 21.0 [14.0, 24.0] | 21.0 [14.0, 24.0] |  |  |
| Sex |  |  |  |  |  |
| Female | 4 (7.0%) | 61 (7.3%) | 65 (7.3%) | 0.00 | 1 |
| Male | 53 (93.0%) | 777 (92.7%) | 830 (92.7%) |  |  |
| Gender |  |  |  |  |  |
| Cisgender | 48 (84.2%) | 676 (80.7%) | 724 (80.9%) | 1.82 | 0.769 |
| Gender diverse Female | 2 (3.5%) | 15 (1.8%) | 17 (1.9%) |  |  |
| Gender diverse Male | 2 (3.5%) | 45 (5.4%) | 47 (5.3%) |  |  |
| Transgender Female | 3 (5.3%) | 56 (6.7%) | 59 (6.6%) |  |  |
| Transgender Male | 2 (3.5%) | 46 (5.5%) | 48 (5.4%) |  |  |
| Sexual orientation |  |  |  |  |  |
| Bisexual | 17 (29.8%) | 217 (25.9%) | 234 (26.1%) | 0.48 | 0.786 |
| Gay/Same Gender Loving/Downe | 30 (52.6%) | 476 (56.8%) | 506 (56.5%) |  |  |
| Other | 10 (17.5%) | 144 (17.2%) | 154 (17.2%) |  |  |
| Missing | 0 (0%) | 1 (0.1%) | 1 (0.1%) |  |  |
| Sexual orientation |  |  |  |  |  |
| Bisexual | 17 (29.8%) | 217 (25.9%) | 234 (26.1%) | 2.08 | 0.838 |
| Gay | 30 (52.6%) | 476 (56.8%) | 506 (56.5%) |  |  |
| Heterosexual | 3 (5.3%) | 38 (4.5%) | 41 (4.6%) |  |  |
| Other non-heterosexual | 1 (1.8%) | 5 (0.6%) | 6 (0.7%) |  |  |
| Pansexual | 3 (5.3%) | 64 (7.6%) | 67 (7.5%) |  |  |
| Queer | 2 (3.5%) | 35 (4.2%) | 37 (4.1%) |  |  |
| Missing | 1 (1.8%) | 3 (0.4%) | 4 (0.4%) |  |  |
| Race |  |  |  |  |  |
| Asian / Pacific Islander | 5 (8.8%) | 48 (5.7%) | 53 (5.9%) | 2.51 | 0.644 |
| Black / African American | 25 (43.9%) | 337 (40.2%) | 362 (40.4%) |  |  |
| Latino | 12 (21.1%) | 245 (29.2%) | 257 (28.7%) |  |  |
| Other | 2 (3.5%) | 37 (4.4%) | 39 (4.4%) |  |  |
| White | 13 (22.8%) | 171 (20.4%) | 184 (20.6%) |  |  |
| City |  |  |  |  |  |
| Los Angeles | 32 (56.1%) | 514 (61.3%) | 546 (61.0%) | 0.41 | 0.523 |
| New Orleans | 25 (43.9%) | 324 (38.7%) | 349 (39.0%) |  |  |
| Education |  |  |  |  |  |
| Below high school | 9 (15.8%) | 128 (15.3%) | 137 (15.3%) | 20.58 | <0.001 |
| High school/equivalent | 26 (45.6%) | 180 (21.5%) | 206 (23.0%) |  |  |
| Some higher education | 17 (29.8%) | 402 (48.0%) | 419 (46.8%) |  |  |
| Completed higher education | 3 (5.3%) | 116 (13.8%) | 119 (13.3%) |  |  |
| Missing | 2 (3.5%) | 12 (1.4%) | 14 (1.6%) |  |  |
| Income above 2021 Federal poverty level |  |  |  |  |  |
| Yes | 12 (21.1%) | 296 (35.3%) | 308 (34.4%) | 4.20 | 0.0403 |
| No | 45 (78.9%) | 542 (64.7%) | 587 (65.6%) |  |  |
| Insurance status |  |  |  |  |  |
| Insured | 42 (73.7%) | 645 (77.0%) | 687 (76.8%) | 0.16 | 0.685 |
| Uninsured/Unsure | 15 (26.3%) | 193 (23.0%) | 208 (23.2%) |  |  |
| What devices owned |  |  |  |  |  |
| Mobile Device | 53 (93.0%) | 812 (96.9%) | 865 (96.6%) | 3.42 | 0.181 |
| No mobile device; has computer | 3 (5.3%) | 15 (1.8%) | 18 (2.0%) |  |  |
| Refuse to answer | 1 (1.8%) | 10 (1.2%) | 11 (1.2%) |  |  |
| Missing | 0 (0%) | 1 (0.1%) | 1 (0.1%) |  |  |
| What devices owned |  |  |  |  |  |
| Mobile device and Computer | 18 (31.6%) | 440 (52.5%) | 458 (51.2%) | 11.29 | 0.0103 |
| Mobile device; No Computer | 35 (61.4%) | 372 (44.4%) | 407 (45.5%) |  |  |
| No mobile device; Has computer | 3 (5.3%) | 15 (1.8%) | 18 (2.0%) |  |  |
| Refuse to answer | 1 (1.8%) | 10 (1.2%) | 11 (1.2%) |  |  |
| Missing | 0 (0%) | 1 (0.1%) | 1 (0.1%) |  |  |
| Amount of access to mobile devices |  |  |  |  |  |
| Borrowed/shared device | 3 (5.3%) | 20 (2.4%) | 23 (2.6%) | 8.09 | 0.0442 |
| No access | 3 (5.3%) | 10 (1.2%) | 13 (1.5%) |  |  |
| Own mobile device | 50 (87.7%) | 794 (94.7%) | 844 (94.3%) |  |  |
| Refuse to answer | 1 (1.8%) | 13 (1.6%) | 14 (1.6%) |  |  |
| Missing | 0 (0%) | 1 (0.1%) | 1 (0.1%) |  |  |
| Amount of access to mobile devices |  |  |  |  |  |
| Can borrow from someone | 2 (3.5%) | 14 (1.7%) | 16 (1.8%) | 13.14 | 0.0688 |
| No access | 3 (5.3%) | 10 (1.2%) | 13 (1.5%) |  |  |
| Own mobile device; minutes and data | 45 (78.9%) | 738 (88.1%) | 783 (87.5%) |  |  |
| Own mobile device; minutes, no data | 5 (8.8%) | 32 (3.8%) | 37 (4.1%) |  |  |
| Refuse to answer | 1 (1.8%) | 13 (1.6%) | 14 (1.6%) |  |  |
| Shared device with friend | 1 (1.8%) | 6 (0.7%) | 7 (0.8%) |  |  |
| Own mobile device; data, no minutes | 0 (0%) | 8 (1.0%) | 8 (0.9%) |  |  |
| Own mobile device; no minutes, no data | 0 (0%) | 16 (1.9%) | 16 (1.8%) |  |  |
| Missing | 0 (0%) | 1 (0.1%) | 1 (0.1%) |  |  |
| Sexually transmitted infection (Lifetime) |  |  |  |  |  |
| Yes | 21 (36.8%) | 287 (34.2%) | 308 (34.4%) | 0.05 | 0.824 |
| No | 36 (63.2%) | 546 (65.2%) | 582 (65.0%) |  |  |
| Missing | 0 (0%) | 5 (0.6%) | 5 (0.6%) |  |  |
| Sexually transmitted infection (Recent) |  |  |  |  |  |
| Yes | 11 (19.3%) | 210 (25.1%) | 221 (24.7%) | 0.69 | 0.406 |
| No | 46 (80.7%) | 625 (74.6%) | 671 (75.0%) |  |  |
| Missing | 0 (0%) | 3 (0.4%) | 3 (0.3%) |  |  |
| Condomless Sex with HIV+ partner (LF) |  |  |  |  |  |
| Yes | 4 (7.0%) | 72 (8.6%) | 76 (8.5%) | 0.03 | 0.867 |
| No | 53 (93.0%) | 766 (91.4%) | 819 (91.5%) |  |  |
| Condomless anal sex in the past year |  |  |  |  |  |
| Yes | 39 (68.4%) | 552 (65.9%) | 591 (66.0%) | 0.02 | 0.88 |
| No | 18 (31.6%) | 278 (33.2%) | 296 (33.1%) |  |  |
| Missing | 0 (0%) | 8 (1.0%) | 8 (0.9%) |  |  |
| 100% Condom Use All Partners (Lifetime) |  |  |  |  |  |
| Yes | 16 (28.1%) | 196 (23.4%) | 212 (23.7%) | 0.39 | 0.533 |
| No | 40 (70.2%) | 623 (74.3%) | 663 (74.1%) |  |  |
| Missing | 1 (1.8%) | 19 (2.3%) | 20 (2.2%) |  |  |
| 100% Condom Use All Partners (Recent) |  |  |  |  |  |
| Yes | 31 (54.4%) | 407 (48.6%) | 438 (48.9%) | 0.46 | 0.497 |
| No | 26 (45.6%) | 427 (51.0%) | 453 (50.6%) |  |  |
| Missing | 0 (0%) | 4 (0.5%) | 4 (0.4%) |  |  |
| No Sexual Activity (past 4 months) |  |  |  |  |  |
| Yes | 7 (12.3%) | 120 (14.3%) | 127 (14.2%) | 0.06 | 0.808 |
| No | 49 (86.0%) | 700 (83.5%) | 749 (83.7%) |  |  |
| Missing | 1 (1.8%) | 18 (2.1%) | 19 (2.1%) |  |  |
| Number of recent sex parters |  |  |  |  |  |
| Mean (SD) | 4.34 (13.4) | 4.25 (12.5) | 4.25 (12.5) | 0.05 | 0.957 |
| Median [Min, Max] | 2.00 [0, 101] | 2.00 [0, 264] | 2.00 [0, 264] |  |  |
| Missing | 1 (1.8%) | 18 (2.1%) | 19 (2.1%) |  |  |
| PrEP Use (Lifetime) |  |  |  |  |  |
| Yes | 7 (12.3%) | 160 (19.1%) | 167 (18.7%) | 1.13 | 0.288 |
| No | 49 (86.0%) | 674 (80.4%) | 723 (80.8%) |  |  |
| Missing | 1 (1.8%) | 4 (0.5%) | 5 (0.6%) |  |  |
| PrEP Use (Recent) |  |  |  |  |  |
| Yes | 3 (5.3%) | 98 (11.7%) | 101 (11.3%) | 1.54 | 0.214 |
| No | 53 (93.0%) | 736 (87.8%) | 789 (88.2%) |  |  |
| Missing | 1 (1.8%) | 4 (0.5%) | 5 (0.6%) |  |  |
| PEP Use (Lifetime) |  |  |  |  |  |
| Yes | 1 (1.8%) | 52 (6.2%) | 53 (5.9%) | 1.15 | 0.283 |
| No | 55 (96.5%) | 780 (93.1%) | 835 (93.3%) |  |  |
| Missing | 1 (1.8%) | 6 (0.7%) | 7 (0.8%) |  |  |
| Completed all doses of PEP Last Time |  |  |  |  |  |
| Yes | 1 (1.8%) | 39 (4.7%) | 40 (4.5%) | 0.00 | 1 |
| No | 0 (0%) | 12 (1.4%) | 12 (1.3%) |  |  |
| Missing | 56 (98.2%) | 787 (93.9%) | 843 (94.2%) |  |  |
| Sex exchange (Lifetime) |  |  |  |  |  |
| Yes | 16 (28.1%) | 201 (24.0%) | 217 (24.2%) | 0.35 | 0.553 |
| No | 40 (70.2%) | 633 (75.5%) | 673 (75.2%) |  |  |
| Missing | 1 (1.8%) | 4 (0.5%) | 5 (0.6%) |  |  |
| Cannabis use (past 4 months) |  |  |  |  |  |
| Yes | 49 (86.0%) | 599 (71.5%) | 648 (72.4%) | 4.74 | 0.0294 |
| No | 8 (14.0%) | 236 (28.2%) | 244 (27.3%) |  |  |
| Missing | 0 (0%) | 3 (0.4%) | 3 (0.3%) |  |  |
| Audit-C Hazardous Drinking |  |  |  |  |  |
| Yes | 20 (35.1%) | 350 (41.8%) | 370 (41.3%) | 0.80 | 0.371 |
| No | 37 (64.9%) | 482 (57.5%) | 519 (58.0%) |  |  |
| Missing | 0 (0%) | 6 (0.7%) | 6 (0.7%) |  |  |
| Opioid Use (past 4 months) |  |  |  |  |  |
| Yes | 4 (7.0%) | 46 (5.5%) | 50 (5.6%) | 0.03 | 0.859 |
| No | 53 (93.0%) | 787 (93.9%) | 840 (93.9%) |  |  |
| Missing | 0 (0%) | 5 (0.6%) | 5 (0.6%) |  |  |
| Stimulants Use (past 4 months) |  |  |  |  |  |
| Yes | 14 (24.6%) | 193 (23.0%) | 207 (23.1%) | 0.02 | 0.888 |
| No | 42 (73.7%) | 638 (76.1%) | 680 (76.0%) |  |  |
| Missing | 1 (1.8%) | 7 (0.8%) | 8 (0.9%) |  |  |
| Poppers use (Lifetime) |  |  |  |  |  |
| Yes | 14 (24.6%) | 246 (29.4%) | 260 (29.1%) | 0.34 | 0.558 |
| No | 42 (73.7%) | 584 (69.7%) | 626 (69.9%) |  |  |
| Missing | 1 (1.8%) | 8 (1.0%) | 9 (1.0%) |  |  |
| Suicide Attempt (lifetime) |  |  |  |  |  |
| Yes | 21 (36.8%) | 246 (29.4%) | 267 (29.8%) | 1.03 | 0.309 |
| No | 35 (61.4%) | 572 (68.3%) | 607 (67.8%) |  |  |
| Missing | 1 (1.8%) | 20 (2.4%) | 21 (2.3%) |  |  |
| PHQ-9 depression symptoms (clinical cutoff) |  |  |  |  |  |
| Yes | 23 (40.4%) | 249 (29.7%) | 272 (30.4%) | 2.33 | 0.127 |
| No | 33 (57.9%) | 571 (68.1%) | 604 (67.5%) |  |  |
| Missing | 1 (1.8%) | 18 (2.1%) | 19 (2.1%) |  |  |
| GAD-7 anxiety symptoms (clinical cutoff) |  |  |  |  |  |
| Yes | 24 (42.1%) | 298 (35.6%) | 322 (36.0%) | 0.61 | 0.436 |
| No | 33 (57.9%) | 529 (63.1%) | 562 (62.8%) |  |  |
| Missing | 0 (0%) | 11 (1.3%) | 11 (1.2%) |  |  |
| Mental Health Hosptialization (Lifetime) |  |  |  |  |  |
| Yes | 21 (36.8%) | 185 (22.1%) | 206 (23.0%) | 5.76 | 0.0164 |
| No | 36 (63.2%) | 653 (77.9%) | 689 (77.0%) |  |  |
| Homelessness (Lifetime) |  |  |  |  |  |
| Yes | 33 (57.9%) | 284 (33.9%) | 317 (35.4%) | 12.42 | <0.001 |
| No | 24 (42.1%) | 554 (66.1%) | 578 (64.6%) |  |  |
| Incarceration (Lifetime) |  |  |  |  |  |
| Yes | 12 (21.1%) | 129 (15.4%) | 141 (15.8%) | 1.00 | 0.318 |
| No | 44 (77.2%) | 706 (84.2%) | 750 (83.8%) |  |  |
| Missing | 1 (1.8%) | 3 (0.4%) | 4 (0.4%) |  |  |
| Interpersonal violence (Lifetime) |  |  |  |  |  |
| Yes | 24 (42.1%) | 249 (29.7%) | 273 (30.5%) | 2.75 | 0.0974 |
| No | 33 (57.9%) | 564 (67.3%) | 597 (66.7%) |  |  |
| Missing | 0 (0%) | 25 (3.0%) | 25 (2.8%) |  |  |
| Service usage (past 4 months) |  |  |  |  |  |
| Yes | 29 (50.9%) | 329 (39.3%) | 358 (40.0%) | 2.47 | 0.116 |
| No | 28 (49.1%) | 506 (60.4%) | 534 (59.7%) |  |  |
| Missing | 0 (0%) | 3 (0.4%) | 3 (0.3%) |  |  |

Supplemental Table 3b. Comparing baseline characteristics of participants with and without follow-up visits in their second year of the study

|  | Has 2nd year follow-up  (N=752) | No 2nd year follow-up  (N=143) | Overall  (N=895) | Chi-Square Test / T-Test Statistic | P-value |
| --- | --- | --- | --- | --- | --- |
| Arm |  |  |  |  |  |
| AMMI | 260 (34.6%) | 53 (37.1%) | 313 (35.0%) | 3.53 | 0.317 |
| AMMI + Coach | 171 (22.7%) | 25 (17.5%) | 196 (21.9%) |  |  |
| AMMI + PS | 175 (23.3%) | 30 (21.0%) | 205 (22.9%) |  |  |
| AMMI + PS + Coach | 146 (19.4%) | 35 (24.5%) | 181 (20.2%) |  |  |
| Age (years) |  |  |  |  |  |
| Mean (SD) | 21.0 (2.16) | 20.9 (2.14) | 21.0 (2.15) | 0.56 | 0.578 |
| Median [Min, Max] | 21.0 [14.0, 24.0] | 21.0 [15.0, 24.0] | 21.0 [14.0, 24.0] |  |  |
| Sex |  |  |  |  |  |
| Female | 56 (7.4%) | 9 (6.3%) | 65 (7.3%) | 0.10 | 0.756 |
| Male | 696 (92.6%) | 134 (93.7%) | 830 (92.7%) |  |  |
| Gender |  |  |  |  |  |
| Cisgender | 609 (81.0%) | 115 (80.4%) | 724 (80.9%) | 1.48 | 0.83 |
| Gender diverse Female | 14 (1.9%) | 3 (2.1%) | 17 (1.9%) |  |  |
| Gender diverse Male | 37 (4.9%) | 10 (7.0%) | 47 (5.3%) |  |  |
| Transgender Female | 50 (6.6%) | 9 (6.3%) | 59 (6.6%) |  |  |
| Transgender Male | 42 (5.6%) | 6 (4.2%) | 48 (5.4%) |  |  |
| Sexual orientation |  |  |  |  |  |
| Bisexual | 192 (25.5%) | 42 (29.4%) | 234 (26.1%) | 3.51 | 0.173 |
| Gay/Same Gender Loving/Downe | 435 (57.8%) | 71 (49.7%) | 506 (56.5%) |  |  |
| Other | 124 (16.5%) | 30 (21.0%) | 154 (17.2%) |  |  |
| Missing | 1 (0.1%) | 0 (0%) | 1 (0.1%) |  |  |
| Sexual orientation |  |  |  |  |  |
| Bisexual | 192 (25.5%) | 42 (29.4%) | 234 (26.1%) | 7.63 | 0.178 |
| Gay | 435 (57.8%) | 71 (49.7%) | 506 (56.5%) |  |  |
| Heterosexual | 33 (4.4%) | 8 (5.6%) | 41 (4.6%) |  |  |
| Other non-heterosexual | 4 (0.5%) | 2 (1.4%) | 6 (0.7%) |  |  |
| Pansexual | 52 (6.9%) | 15 (10.5%) | 67 (7.5%) |  |  |
| Queer | 34 (4.5%) | 3 (2.1%) | 37 (4.1%) |  |  |
| Missing | 2 (0.3%) | 2 (1.4%) | 4 (0.4%) |  |  |
| Race |  |  |  |  |  |
| Asian / Pacific Islander | 47 (6.3%) | 6 (4.2%) | 53 (5.9%) | 8.48 | 0.0756 |
| Black / African American | 297 (39.5%) | 65 (45.5%) | 362 (40.4%) |  |  |
| Latino | 228 (30.3%) | 29 (20.3%) | 257 (28.7%) |  |  |
| Other | 33 (4.4%) | 6 (4.2%) | 39 (4.4%) |  |  |
| White | 147 (19.5%) | 37 (25.9%) | 184 (20.6%) |  |  |
| City |  |  |  |  |  |
| Los Angeles | 464 (61.7%) | 82 (57.3%) | 546 (61.0%) | 0.79 | 0.375 |
| New Orleans | 288 (38.3%) | 61 (42.7%) | 349 (39.0%) |  |  |
| Education |  |  |  |  |  |
| Below high school | 111 (14.8%) | 26 (18.2%) | 137 (15.3%) | 24.74 | <0.001 |
| High school/equivalent | 153 (20.3%) | 53 (37.1%) | 206 (23.0%) |  |  |
| Some higher education | 369 (49.1%) | 50 (35.0%) | 419 (46.8%) |  |  |
| Completed higher education | 108 (14.4%) | 11 (7.7%) | 119 (13.3%) |  |  |
| Missing | 11 (1.5%) | 3 (2.1%) | 14 (1.6%) |  |  |
| Income above 2021 Federal poverty level |  |  |  |  |  |
| Yes | 267 (35.5%) | 41 (28.7%) | 308 (34.4%) | 2.19 | 0.139 |
| No | 485 (64.5%) | 102 (71.3%) | 587 (65.6%) |  |  |
| Insurance status |  |  |  |  |  |
| Insured | 582 (77.4%) | 105 (73.4%) | 687 (76.8%) | 0.85 | 0.357 |
| Uninsured/Unsure | 170 (22.6%) | 38 (26.6%) | 208 (23.2%) |  |  |
| What devices owned |  |  |  |  |  |
| Mobile Device | 731 (97.2%) | 134 (93.7%) | 865 (96.6%) | 5.23 | 0.0731 |
| No mobile device; has computer | 12 (1.6%) | 6 (4.2%) | 18 (2.0%) |  |  |
| Refuse to answer | 8 (1.1%) | 3 (2.1%) | 11 (1.2%) |  |  |
| Missing | 1 (0.1%) | 0 (0%) | 1 (0.1%) |  |  |
| What devices owned |  |  |  |  |  |
| Mobile device and Computer | 399 (53.1%) | 59 (41.3%) | 458 (51.2%) | 10.16 | 0.0172 |
| Mobile device; No Computer | 332 (44.1%) | 75 (52.4%) | 407 (45.5%) |  |  |
| No mobile device; Has computer | 12 (1.6%) | 6 (4.2%) | 18 (2.0%) |  |  |
| Refuse to answer | 8 (1.1%) | 3 (2.1%) | 11 (1.2%) |  |  |
| Missing | 1 (0.1%) | 0 (0%) | 1 (0.1%) |  |  |
| Amount of access to mobile devices |  |  |  |  |  |
| Borrowed/shared device | 16 (2.1%) | 7 (4.9%) | 23 (2.6%) | 10.66 | 0.0137 |
| No access | 8 (1.1%) | 5 (3.5%) | 13 (1.5%) |  |  |
| Own mobile device | 717 (95.3%) | 127 (88.8%) | 844 (94.3%) |  |  |
| Refuse to answer | 10 (1.3%) | 4 (2.8%) | 14 (1.6%) |  |  |
| Missing | 1 (0.1%) | 0 (0%) | 1 (0.1%) |  |  |
| Amount of access to mobile devices |  |  |  |  |  |
| Can borrow from someone | 11 (1.5%) | 5 (3.5%) | 16 (1.8%) | 12.16 | 0.0955 |
| No access | 8 (1.1%) | 5 (3.5%) | 13 (1.5%) |  |  |
| Own mobile device; data, no minutes | 7 (0.9%) | 1 (0.7%) | 8 (0.9%) |  |  |
| Own mobile device; minutes and data | 668 (88.8%) | 115 (80.4%) | 783 (87.5%) |  |  |
| Own mobile device; minutes, no data | 29 (3.9%) | 8 (5.6%) | 37 (4.1%) |  |  |
| Own mobile device; no minutes, no data | 13 (1.7%) | 3 (2.1%) | 16 (1.8%) |  |  |
| Refuse to answer | 10 (1.3%) | 4 (2.8%) | 14 (1.6%) |  |  |
| Shared device with friend | 5 (0.7%) | 2 (1.4%) | 7 (0.8%) |  |  |
| Missing | 1 (0.1%) | 0 (0%) | 1 (0.1%) |  |  |
| Sexually transmitted infection (Lifetime) |  |  |  |  |  |
| Yes | 264 (35.1%) | 44 (30.8%) | 308 (34.4%) | 0.80 | 0.372 |
| No | 484 (64.4%) | 98 (68.5%) | 582 (65.0%) |  |  |
| Missing | 4 (0.5%) | 1 (0.7%) | 5 (0.6%) |  |  |
| Sexually transmitted infection (Recent) |  |  |  |  |  |
| Yes | 190 (25.3%) | 31 (21.7%) | 221 (24.7%) | 0.61 | 0.435 |
| No | 560 (74.5%) | 111 (77.6%) | 671 (75.0%) |  |  |
| Missing | 2 (0.3%) | 1 (0.7%) | 3 (0.3%) |  |  |
| Condomless Sex with HIV+ partner (LF) |  |  |  |  |  |
| Yes | 64 (8.5%) | 12 (8.4%) | 76 (8.5%) | 0.00 | 1 |
| No | 688 (91.5%) | 131 (91.6%) | 819 (91.5%) |  |  |
| Condomless anal sex in the past year |  |  |  |  |  |
| Yes | 494 (65.7%) | 97 (67.8%) | 591 (66.0%) | 0.13 | 0.714 |
| No | 251 (33.4%) | 45 (31.5%) | 296 (33.1%) |  |  |
| Missing | 7 (0.9%) | 1 (0.7%) | 8 (0.9%) |  |  |
| 100% Condom Use All Partners (Lifetime) |  |  |  |  |  |
| Yes | 171 (22.7%) | 41 (28.7%) | 212 (23.7%) | 1.85 | 0.174 |
| No | 563 (74.9%) | 100 (69.9%) | 663 (74.1%) |  |  |
| Missing | 18 (2.4%) | 2 (1.4%) | 20 (2.2%) |  |  |
| 100% Condom Use All Partners (Recent) |  |  |  |  |  |
| Yes | 360 (47.9%) | 78 (54.5%) | 438 (48.9%) | 1.73 | 0.188 |
| No | 388 (51.6%) | 65 (45.5%) | 453 (50.6%) |  |  |
| Missing | 4 (0.5%) | 0 (0%) | 4 (0.4%) |  |  |
| No Sexual Activity (past 4 months) |  |  |  |  |  |
| Yes | 107 (14.2%) | 20 (14.0%) | 127 (14.2%) | 0.00 | 1 |
| No | 631 (83.9%) | 118 (82.5%) | 749 (83.7%) |  |  |
| Missing | 14 (1.9%) | 5 (3.5%) | 19 (2.1%) |  |  |
| Number of recent sex parters |  |  |  |  |  |
| Mean (SD) | 4.21 (13.0) | 4.49 (9.43) | 4.25 (12.5) | -0.25 | 0.806 |
| Median [Min, Max] | 2.00 [0, 264] | 2.00 [0, 101] | 2.00 [0, 264] |  |  |
| Missing | 14 (1.9%) | 5 (3.5%) | 19 (2.1%) |  |  |
| PrEP Use (Lifetime) |  |  |  |  |  |
| Yes | 153 (20.3%) | 14 (9.8%) | 167 (18.7%) | 8.11 | 0.00441 |
| No | 595 (79.1%) | 128 (89.5%) | 723 (80.8%) |  |  |
| Missing | 4 (0.5%) | 1 (0.7%) | 5 (0.6%) |  |  |
| PrEP Use (Recent) |  |  |  |  |  |
| Yes | 97 (12.9%) | 4 (2.8%) | 101 (11.3%) | 11.24 | <0.001 |
| No | 651 (86.6%) | 138 (96.5%) | 789 (88.2%) |  |  |
| Missing | 4 (0.5%) | 1 (0.7%) | 5 (0.6%) |  |  |
| PEP Use (Lifetime) |  |  |  |  |  |
| Yes | 48 (6.4%) | 5 (3.5%) | 53 (5.9%) | 1.28 | 0.258 |
| No | 699 (93.0%) | 136 (95.1%) | 835 (93.3%) |  |  |
| Missing | 5 (0.7%) | 2 (1.4%) | 7 (0.8%) |  |  |
| Completed all doses of PEP Last Time |  |  |  |  |  |
| Yes | 36 (4.8%) | 4 (2.8%) | 40 (4.5%) | 0.00 | 1 |
| No | 11 (1.5%) | 1 (0.7%) | 12 (1.3%) |  |  |
| Missing | 705 (93.8%) | 138 (96.5%) | 843 (94.2%) |  |  |
| Sex exchange (Lifetime) |  |  |  |  |  |
| Yes | 180 (23.9%) | 37 (25.9%) | 217 (24.2%) | 0.16 | 0.689 |
| No | 568 (75.5%) | 105 (73.4%) | 673 (75.2%) |  |  |
| Missing | 4 (0.5%) | 1 (0.7%) | 5 (0.6%) |  |  |
| Cannabis use (past 4 months) |  |  |  |  |  |
| Yes | 530 (70.5%) | 118 (82.5%) | 648 (72.4%) | 7.77 | 0.00531 |
| No | 219 (29.1%) | 25 (17.5%) | 244 (27.3%) |  |  |
| Missing | 3 (0.4%) | 0 (0%) | 3 (0.3%) |  |  |
| Audit-C Hazardous Drinking |  |  |  |  |  |
| Yes | 318 (42.3%) | 52 (36.4%) | 370 (41.3%) | 1.33 | 0.249 |
| No | 430 (57.2%) | 89 (62.2%) | 519 (58.0%) |  |  |
| Missing | 4 (0.5%) | 2 (1.4%) | 6 (0.7%) |  |  |
| Opioid Use (past 4 months) |  |  |  |  |  |
| Yes | 41 (5.5%) | 9 (6.3%) | 50 (5.6%) | 0.04 | 0.835 |
| No | 707 (94.0%) | 133 (93.0%) | 840 (93.9%) |  |  |
| Missing | 4 (0.5%) | 1 (0.7%) | 5 (0.6%) |  |  |
| Stimulants Use (past 4 months) |  |  |  |  |  |
| Yes | 174 (23.1%) | 33 (23.1%) | 207 (23.1%) | 0.00 | 1 |
| No | 573 (76.2%) | 107 (74.8%) | 680 (76.0%) |  |  |
| Missing | 5 (0.7%) | 3 (2.1%) | 8 (0.9%) |  |  |
| Poppers use (Lifetime) |  |  |  |  |  |
| Yes | 222 (29.5%) | 38 (26.6%) | 260 (29.1%) | 0.34 | 0.562 |
| No | 523 (69.5%) | 103 (72.0%) | 626 (69.9%) |  |  |
| Missing | 7 (0.9%) | 2 (1.4%) | 9 (1.0%) |  |  |
| Suicide Attempt (lifetime) |  |  |  |  |  |
| Yes | 224 (29.8%) | 43 (30.1%) | 267 (29.8%) | 0.00 | 0.994 |
| No | 511 (68.0%) | 96 (67.1%) | 607 (67.8%) |  |  |
| Missing | 17 (2.3%) | 4 (2.8%) | 21 (2.3%) |  |  |
| PHQ-9 depression symptoms (clinical cutoff) |  |  |  |  |  |
| Yes | 219 (29.1%) | 53 (37.1%) | 272 (30.4%) | 3.24 | 0.072 |
| No | 517 (68.8%) | 87 (60.8%) | 604 (67.5%) |  |  |
| Missing | 16 (2.1%) | 3 (2.1%) | 19 (2.1%) |  |  |
| GAD-7 anxiety symptoms (clinical cutoff) |  |  |  |  |  |
| Yes | 263 (35.0%) | 59 (41.3%) | 322 (36.0%) | 2.06 | 0.151 |
| No | 481 (64.0%) | 81 (56.6%) | 562 (62.8%) |  |  |
| Missing | 8 (1.1%) | 3 (2.1%) | 11 (1.2%) |  |  |
| Mental Health Hosptialization (Lifetime) |  |  |  |  |  |
| Yes | 163 (21.7%) | 43 (30.1%) | 206 (23.0%) | 4.32 | 0.0378 |
| No | 589 (78.3%) | 100 (69.9%) | 689 (77.0%) |  |  |
| Homelessness (Lifetime) |  |  |  |  |  |
| Yes | 241 (32.0%) | 76 (53.1%) | 317 (35.4%) | 22.47 | <0.001 |
| No | 511 (68.0%) | 67 (46.9%) | 578 (64.6%) |  |  |
| Incarceration (Lifetime) |  |  |  |  |  |
| Yes | 105 (14.0%) | 36 (25.2%) | 141 (15.8%) | 10.68 | 0.00109 |
| No | 644 (85.6%) | 106 (74.1%) | 750 (83.8%) |  |  |
| Missing | 3 (0.4%) | 1 (0.7%) | 4 (0.4%) |  |  |
| Interpersonal violence (Lifetime) |  |  |  |  |  |
| Yes | 220 (29.3%) | 53 (37.1%) | 273 (30.5%) | 3.38 | 0.0659 |
| No | 512 (68.1%) | 85 (59.4%) | 597 (66.7%) |  |  |
| Missing | 20 (2.7%) | 5 (3.5%) | 25 (2.8%) |  |  |
| Service usage (past 4 months) |  |  |  |  |  |
| Yes | 284 (37.8%) | 74 (51.7%) | 358 (40.0%) | 8.99 | 0.00271 |
| No | 465 (61.8%) | 69 (48.3%) | 534 (59.7%) |  |  |
| Missing | 3 (0.4%) | 0 (0%) | 3 (0.3%) |  |  |

**Online Resource 3: Sensitivity Analysis of City Effects on Intervention effects on Support Services Utilization**

To investigate the possibility intervention effect modification by city for the support services outcome, we fit a Generalized Linear Mixed Model (GLMM) with a random intercept for each participant that included a three-way interaction between the intervention arm, the time variable (visit), and city. The model was fit using the “MASS” library in R using penalized quasi-likelihood (PQL). The reference group is the automated messaging and monitoring intervention (AMMI) condition in Los Angeles, and the odds ratios (OR), 95% confidence intervals, and the Bonferroni corrected 99.17% confidence intervals (based on α = .05 / 6 = .0083) are presented.

Supplemental Table 4 below shows results. Although we do see significant baseline differences of service usage across arms and cities, none of the three-way interactions between arms, the linear time trend of visit, and city are significant. This indicates that there are no significant differences between cities in the interventions’ effects on service usage over time. Furthermore, even when accounting for possible differences between cities, we still see the significant effect of the two coaching intervention arms on support service usage over time.

Supplemental Table 4: Results of a Generalized Linear Mixed Model (GLMM) with random intercept for each participant and a three-way interaction between city, arm, and visit over 24 months follow-up (n=895).

| **Variable** | **Odds Ratio (OR) Estimate** | **95% Confidence Interval** | **99.17% Confidence Interval** |
| --- | --- | --- | --- |
| Intercept | 2.79 | 1.87 - 4.16* | 1.62 - 4.79* |
| AMMI+Coaching (C) | 0.15 | 0.08 - 0.29* | 0.06 - 0.36* |
| AMMI+Peer Support (PS) | 0.27 | 0.15 - 0.51* | 0.12 - 0.63* |
| AMMI+PS+C | 0.17 | 0.09 - 0.32* | 0.07 - 0.40* |
| City - New Orleans [Ref city = Los Angeles] | 0.08 | 0.04 - 0.14* | 0.03 - 0.18* |
| AMMI+C × New Orleans | 2.43 | 0.83 - 7.14 | 0.57 - 10.36 |
| AMMI+PS × New Orleans | 2.76 | 0.99 - 7.70 | 0.69 - 10.98 |
| AMMI+PS+C × New Orleans | 3.20 | 1.09 - 9.39* | 0.75 - 13.62 |
| Visit | 0.71 | 0.67 - 0.77* | 0.65 - 0.79* |
| Visit × AMMI+C | 1.22 | 1.09 - 1.36* | 1.05 - 1.42* |
| Visit × AMMI+PS | 0.99 | 0.89 - 1.11 | 0.85 - 1.16 |
| Visit × PS+C | 1.19 | 1.06 - 1.34* | 1.02 - 1.40* |
| Visit × New Orleans | 1.07 | 0.95 - 1.20 | 0.91 - 1.26 |
| Visit × AMMI+C × New Orleans | 1.07 | 0.88 - 1.31 | 0.82 - 1.40 |
| Visit × AMMI+PS × New Orleans | 1.14 | 0.94 - 1.38 | 0.88 - 1.47 |
| Visit × AMMI+PS+C × New Orleans | 1.11 | 0.92 - 1.35 | 0.86 - 1.44 |

Note: the reference group is the AMMI-only condition in Los Angeles

**Online Resources 4 to 6**

**Supplemental Utilization Outcome Analyses**

**Methodology**

Generalized linear mixed models (GLMMs) were fit with main effects only and with covariate adjustments with the same covariates as in the main analyses. PQL (MASS:glmmPQL) was used to fit the health care provider model with binary outcomes (Figure 5, Table 5). For the outcome on mental health specialist visits (Figure 6, Table 6) due to large outliers, the data was winsorized at 20 based on distribution of data and to reflect approximately weekly counseling appointments over 4 months or 17 weeks. The over-dispersion in the data led us to use a negative binomial model with linear time trend that was fin using Bayesian methods (brms). For outcomes on mental health support group (Figure 7, Table 7) and HIV prevention program participation (Figure 8, Table 8) there were also large outliers, so again, the data was also winsorized at 20. In addition to this, there was significant over-dispersion at baseline along with nonlinearity. A negative binomial model along with a quadratic visit term was fit for these outcomes using Bayesian methods (brms). The Bayesian approach was used for these outcomes because of computational difficulties in fitting the frequentist models. Adjustment for multiple testing was done by applying a Bonferroni correction to the interval estimates based on the number of outcomes. Since there were 6 utilization outcomes analyzed, the corrected alpha is *α* = 0*.*05*/*6 = 0*.*0083. The adjusted regression models had the following covariates: age, gender, race, sexual orientation, city, have insurance, income, lifetime homelessness, lifetime incarceration, lifetime sex exchange, lifetime mental health hospitalization, after COVID stay at home orders (3/17/20), enrollment time, and mobile phone access.

## Supplemental Figure 5: Observed proportions of participants who have a regular healthcare provider in the past 4 months over 24 months by study arm with linear trend (n = 895).


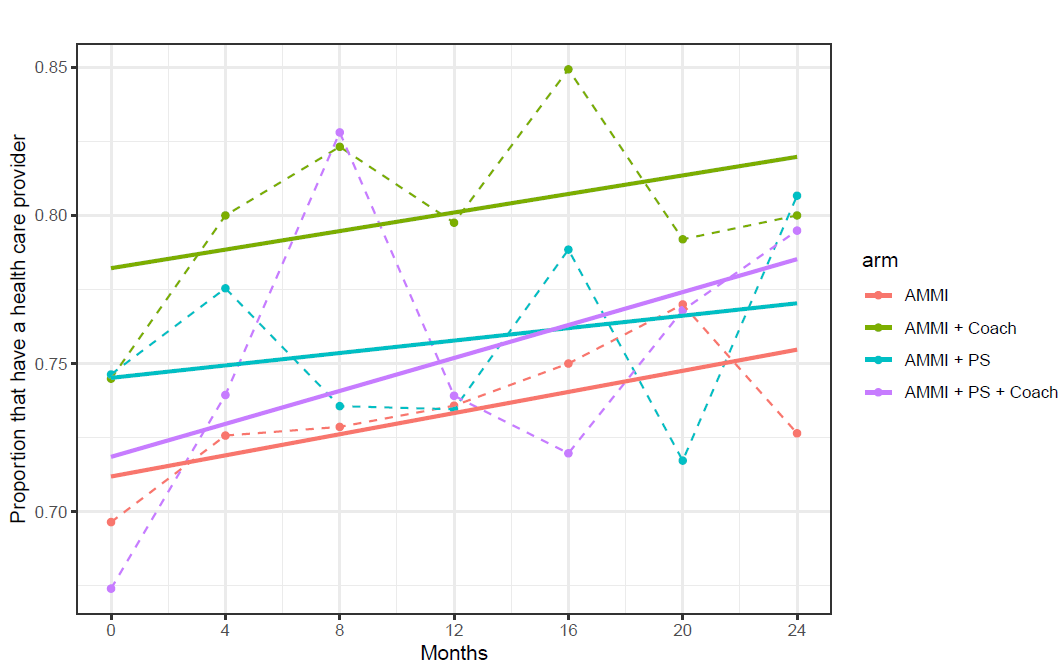


## Supplemental Table 5: Estimated intervention effects for having a regular healthcare provider in the past 4 months over 24 months without covariate adjustment (N = 894), and with covariate adjustment (N = 886).

|  |  |  |  | Adjusted Model with Covariates (N = 886) | | |
| --- | --- | --- | --- | --- | --- | --- |
| Variable | Odds Ratio Estimate | 95% Confidence Interval | 99.17% Confidence Interval^ | Odds Ratio Estimate | 95% Confidence Interval | 99.17% Confidence Interval^ |
| Intercept | 3.73 | 2.85 - 4.89* | 2.60 - 5.37* | 16.25 | 3.41 - 77.55* | 1.98 - 133.18* |
| AMMI + Coach | 1.56 | 1.00 - 2.44 | 0.85 - 2.86 | 1.61 | 1.01 - 2.56* | 0.86 - 3.01 |
| AMMI + PS | 1.37 | 0.89 - 2.12 | 0.76 - 2.46 | 1.17 | 0.75 - 1.81 | 0.64 - 2.11 |
| AMMI + PS + Coach | 0.96 | 0.62 - 1.50 | 0.53 - 1.76 | 0.82 | 0.52 - 1.28 | 0.45 - 1.5 |
| Visit | 1.01 | 0.95 - 1.06 | 0.94 - 1.08 | 1.03 | 0.97 - 1.09 | 0.95 - 1.12 |
| Visit x AMMI + Coach | 1.03 | 0.94 - 1.13 | 0.92 - 1.16 | 0.98 | 0.89 - 1.08 | 0.86 - 1.11 |
| Visit x armAMMI + PS | 0.96 | 0.88 - 1.04 | 0.86 - 1.07 | 0.95 | 0.87 - 1.04 | 0.84 - 1.07 |
| Visit x armAMMI + PS + Coach | 1.09 | 1.00 - 1.19 | 0.97 - 1.22 | 1.08 | 0.99 - 1.19 | 0.96 - 1.23 |

*: confidence interval does not include 1

^: Bonferroni corrected confidence intervals based on alpha = .05 / 6 = .0083 for four outcomes

## Supplemental Figure 6: Mean number of times received care from a mental health specialist in the past 4 months over 24 months across intervention condition


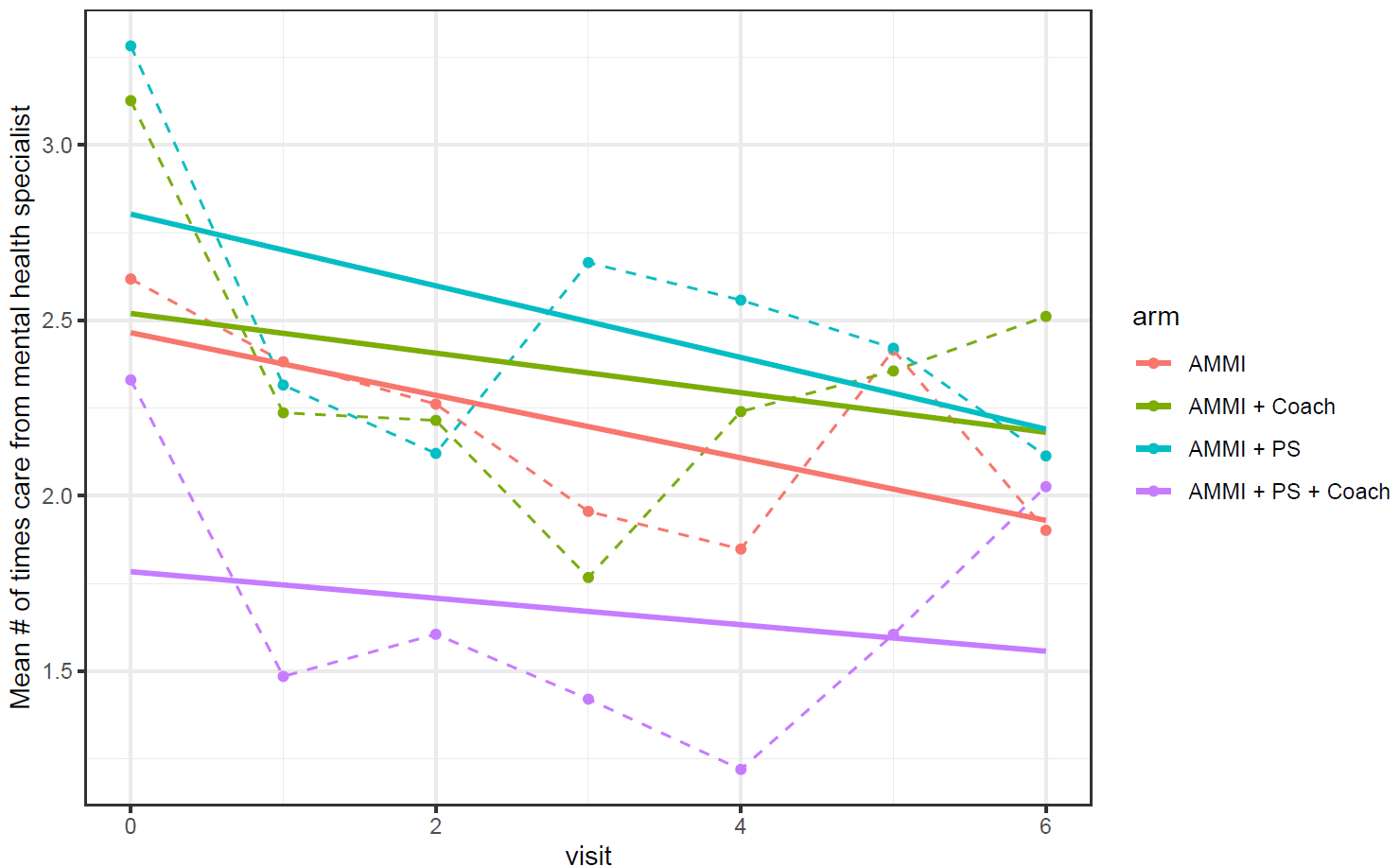


## Supplemental Table 6: Results of General Linear Mixed Model (GLMM) regression model comparing intervention condition slopes over time to automated messaging and monitoring intervention (AMMI) only for number of times receiving care from a mental health specialist in the past four months (n=895), and adjusted model controlling for baseline covariates (N=886).

|  |  |  |  | Adjusted Model with Covariates (N = 882) | | |
| --- | --- | --- | --- | --- | --- | --- |
| Variable | Incidence Rate Ratio Estimate | 95% Confidence Interval | 99.17% Confidence Interval^ | Incidence Rate Ratio Estimate | 95% Confidence Interval | 99.17% Confidence Interval^ |
| Intercept | 0.48 | 0.33 - 0.65* | 0.29 - 0.77* | 0.87 | 0.11 - 4.75 | 0.06 – 12.00 |
| AMMI + Coach | 0.73 | 0.40 - 1.19 | 0.33 - 1.59 | 0.79 | 0.44 - 1.29 | 0.35 - 1.75 |
| AMMI + PS | 0.99 | 0.56 - 1.59 | 0.44 - 2.09 | 1.18 | 0.67 - 1.86 | 0.55 - 2.49 |
| AMMI + PS + Coach | 0.59 | 0.32 - 0.99* | 0.26 - 1.33 | 0.67 | 0.38 - 1.10 | 0.31 - 1.50 |
| Visit | 0.90 | 0.85 - 0.95* | 0.83 - 0.98* | 0.90 | 0.84 - 0.96* | 0.82 - 0.99* |
| Visit x AMMI + Coach | 1.08 | 0.98 - 1.18 | 0.95 - 1.24 | 1.07 | 0.97 - 1.17 | 0.94 - 1.24 |
| Visit x armAMMI + PS | 1.04 | 0.95 - 1.13 | 0.91 - 1.19 | 1.05 | 0.95 - 1.14 | 0.92 - 1.19 |
| Visit x armAMMI + PS + Coach | 1.07 | 0.96 - 1.17 | 0.93 - 1.24 | 1.10 | 0.99 - 1.20 | 0.95 - 1.27 |

Note: Adjusted model controls for baseline covariates shown in Table 4

^ Bonferroni corrected 99.17% confidence intervals for six outcomes (based on alpha = .05 / 6 = 0.0083)

* indicates that the confidence interval does not contain 1

## Supplemental Figure 7: Observed mean number of times received care from mental health specialist support group in past 4 months over 24 month by study arm with linear trends


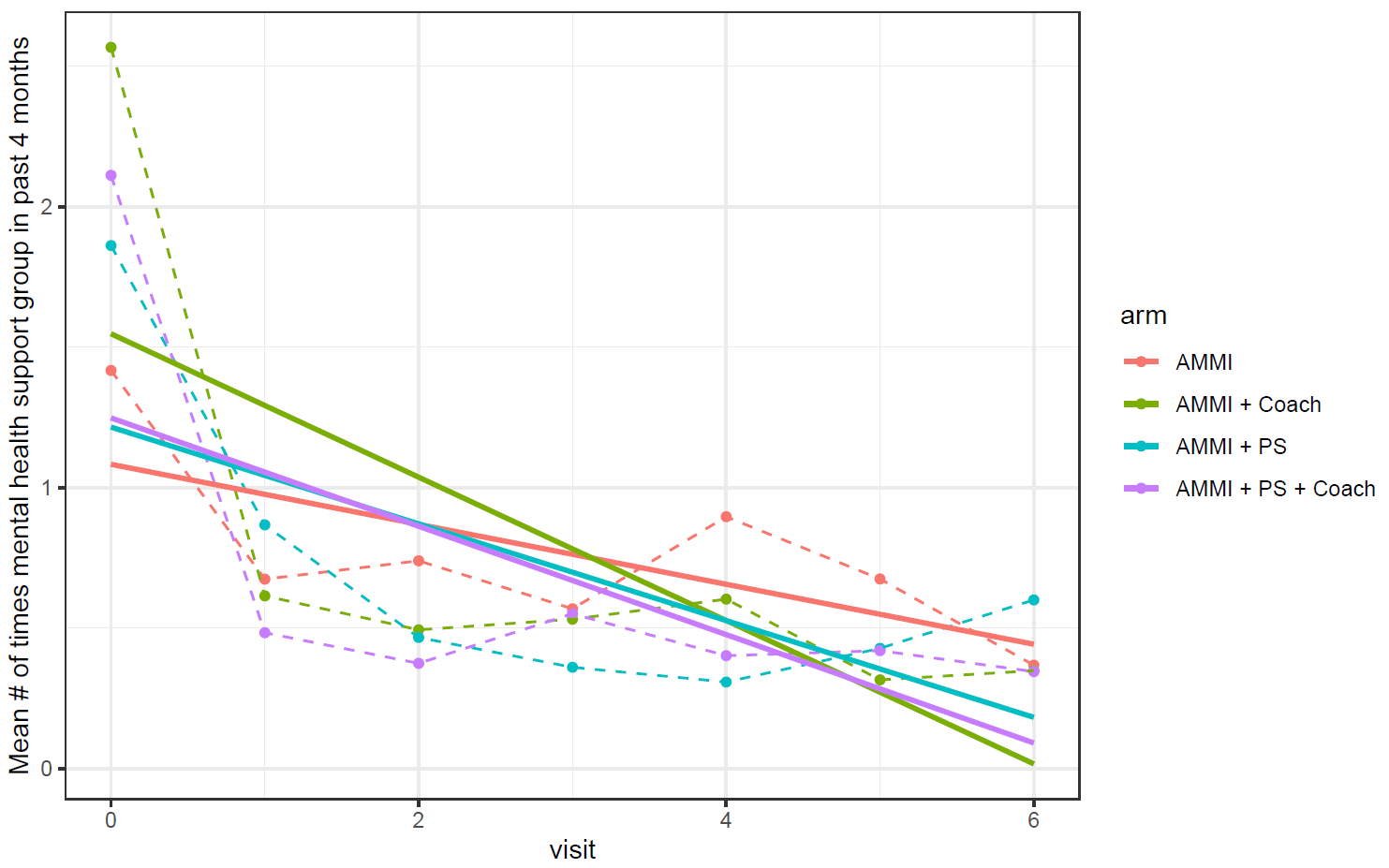


## Supplemental Table 7: Estimated intervention effects for mental health support group care in past 4 months over 24 months, with (n = 881) and without (n = 873) covariate adjustments

|  |  |  |  | Adjusted Model with Covariates (N = 882) | | |
| --- | --- | --- | --- | --- | --- | --- |
| Variable | Incidence Rate Ratio Estimate | 95% Confidence Interval | 99.17% Confidence Interval^ | Incidence Rate Ratio Estimate | 95% Confidence Interval | 99.17% Confidence Interval^ |
| Intercept | 0.13 | 0.05 - 0.29* | 0.03 - 0.50* | 0.01 | 0.00 - 0.23* | 0.00 - 1.58 |
| AMMI + Coach | 0.98 | 0.29 - 2.75 | 0.19 - 4.95 | 1.39 | 0.37 - 3.91 | 0.24 - 7.15 |
| AMMI + PS | 0.72 | 0.21 - 1.96 | 0.15 - 3.42 | 1.06 | 0.30 - 3.06 | 0.19 - 6.02 |
| AMMI + PS + Coach | 0.31 | 0.08 - 0.92* | 0.05 - 1.86 | 0.44 | 0.11 - 1.41 | 0.06 - 2.70 |
| Visit | 0.47 | 0.29 - 0.68* | 0.24 - 0.83* | 0.44 | 0.28 - 0.65* | 0.24 - 0.8* |
| Visit x AMMI + Coach | 0.98 | 0.73 - 1.25 | 0.66 - 1.44 | 0.97 | 0.73 - 1.25 | 0.66 - 1.44 |
| Visit x armAMMI + PS | 0.99 | 0.75 - 1.26 | 0.69 - 1.45 | 1.02 | 0.77 - 1.31 | 0.68 - 1.50 |
| Visit x armAMMI + PS + Coach | 1.13 | 0.82 - 1.47 | 0.74 - 1.73 | 1.15 | 0.83 - 1.51 | 0.75 - 1.81 |
| Visit^2^ | 1.08 | 1.02 - 1.14* | 1.00 - 1.18 | 1.09 | 1.02 - 1.15* | 1.01 - 1.19* |

Note: Adjusted model controls for baseline covariates shown in Table 4

^ Bonferroni corrected 99.17% confidence intervals for six outcomes (based on alpha = .05 / 6 = 0.0083)

* indicates that the confidence interval does not contain 1

## Supplemental Figure 8: Observed Mean number of times participated in a HIV prevention program in the past 4 months over 24 months by study arm with linear trend (N = 895)


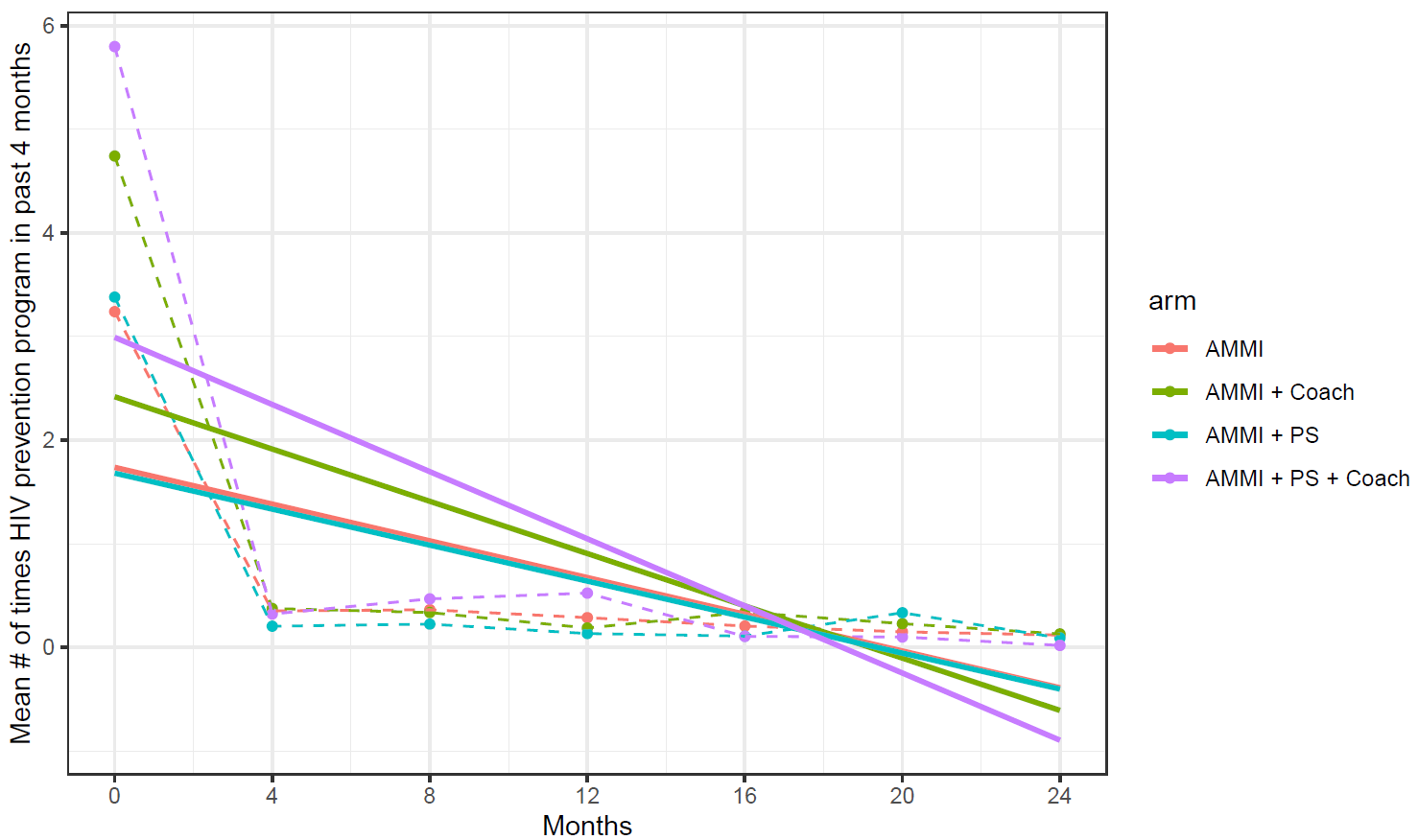


## Supplemental Table 8: Estimated intervention effects for HIV prevention program participation in past 4 months over 24 months, without covariate adjustment (n = 890), and with covariate adjustment (n = 882).

|  |  |  |  | Adjusted Model with Covariates (N = 882) | | |
| --- | --- | --- | --- | --- | --- | --- |
| Variable | Incidence Rate Ratio Estimate | 95% Confidence Interval | 99.17% Confidence Interval^ | Incidence Rate Ratio Estimate | 95% Confidence Interval | 99.17% Confidence Interval^ |
| Intercept | 0.89 | 0.61 - 1.20 | 0.54 - 1.44 | 0.08 | 0.01 - 0.4* | 0.01 - 1.05 |
| AMMI + Coach | 0.87 | 0.51 - 1.36 | 0.43 - 1.78 | 1.59 | 0.90 - 2.58 | 0.74 - 3.39 |
| AMMI + PS | 0.57 | 0.33 - 0.90* | 0.27 - 1.20 | 1.06 | 0.61 - 1.68 | 0.51 - 2.26 |
| AMMI + PS + Coach | 1.38 | 0.78 - 2.17 | 0.66 - 2.98 | 2.37 | 1.33 - 3.82* | 1.10 - 5.04* |
| Visit | 0.24 | 0.20 - 0.28* | 0.19 - 0.31* | 0.24 | 0.20 - 0.29* | 0.19 - 0.31* |
| Visit x AMMI + Coach | 1.04 | 0.91 - 1.17 | 0.86 - 1.25 | 1.05 | 0.91 - 1.18 | 0.86 - 1.27 |
| Visit x armAMMI + PS | 1.06 | 0.93 - 1.19 | 0.88 - 1.28 | 1.08 | 0.93 - 1.21 | 0.89 - 1.30 |
| Visit x armAMMI + PS + Coach | 0.84 | 0.72 - 0.96* | 0.67 - 1.04 | 0.86 | 0.74 - 0.99* | 0.70 - 1.07 |
| Visit^2^ | 1.17 | 1.14 - 1.2* | 1.13 - 1.22* | 1.17 | 1.13 - 1.20* | 1.12 - 1.22* |

*: confidence interval does not include 1

^: Bonferroni corrected confidence intervals based on (alpha = .05 / 6 = .0083)
